# Health Literacy Needs Among Unemployed Persons: Collating Evidence Through Triangulation of Interview and Scoping Review Data

**DOI:** 10.3389/fpubh.2022.798797

**Published:** 2022-02-22

**Authors:** Florence Samkange-Zeeb, Hunny Singh, Meret Lakeberg, Jonathan Kolschen, Benjamin Schüz, Lara Christianson, Karina Karolina De Santis, Tilman Brand, Hajo Zeeb

**Affiliations:** ^1^Department of Prevention and Evaluation, Leibniz Institute for Prevention Research and Epidemiology – BIPS, Bremen, Germany; ^2^Faculty of Human and Health Sciences (Public Health), University of Bremen, Bremen, Germany

**Keywords:** health literacy (MeSH), needs assessment [MeSH], unemployed, scoping review, semi-structured interviews, participatory research

## Abstract

**Background:**

Unemployed persons are at high risk for low health literacy. Most studies addressing health literacy of unemployed persons focus on risk factors for low health literacy or correlates of health literacy, but studies on needs of unemployed persons regarding health literacy are scarce. We aimed to obtain better understanding of health literacy needs of unemployed adults by triangulating the results from a scoping review on health literacy needs in unemployed adults and additional in-depth qualitative interviews.

**Methods:**

Scoping review: We searched six databases up to January 2021 as well as gray literature for relevant studies following PRISMA-ScR guidelines. Titles, abstracts, and full texts were screened independently by two researchers. Qualitative study: Ten participants of a job-reintegration program in Germany were interviewed following a guideline covering topics including health issues of interest to the participants, their sources of health-related information and the barriers/facilitators they experience when accessing health services.

**Results:**

Scoping review: After screening 2,966 titles and abstracts, 36 full texts were considered, and five articles fulfilled the inclusion criteria. Four focused on mental health literacy and outcomes, while the fifth assessed information-seeking practices. One additional report on health literacy was identified via the gray literature search. Awareness of one's condition was identified as a facilitator for mental health help-seeking, while fear of harmful effects of medication prevented help-seeking. Qualitative study: Participants were interested in and were generally well-informed about health topics such as nutrition and physical activity. The main challenge perceived was translating the knowledge into practice in daily life. GPs and the social services providers played an important role as a source of health information and advice. Regarding mental health, similar barriers, facilitators and needs were identified through triangulation of findings of the scoping review with those of the interviews.

**Conclusions:**

There is need to address health literacy needs of long-term unemployed persons that go beyond mental health literacy. Public health interventions should not only aim at improving health literacy scores, but also focus on how to help participants translate health literacy into practice. Population groups of interest should also be involved in all processes of designing interventions.

## Introduction

Health literacy is often defined as “the knowledge, motivation and competences to access, understand, appraise and apply health information in order to make judgements and take decisions in everyday life concerning health care, disease prevention and health promotion to maintain or improve quality of life throughout the course of life” (1). However, different definitions exist: While this definition centers on capacities of the individual in the decision-making process, others highlight the importance of the social environment for health literacy (2).

Nevertheless, the concept is evolving and continues to gain importance globally (3). It has been included in many policy programs such as the United States' Healthy People 2030 initiative (2) or Germany's National Action Plan Health Literacy (4). It has been suggested that persons with low health literacy suffer from poorer overall health (5) and find it more difficult to follow doctors' instructions, or take medication as prescribed (6) compared to those with higher health literacy scores. They have also been reported to use hospital and out-patient services more and to use preventive measures less, thereby incurring more medical costs (7–9). Because health literacy affects many areas of life, it has been argued that differences in health literacy can cause or exacerbate health inequalities (10, 11). A large-scale European survey including 8,000 individuals from eight countries found more than a tenth of the whole sample (12.4%) to have inadequate health literacy (12). The proportion however varied between countries and ranged from 1.6% in the Netherlands to 26.9% in Bulgaria. The respective proportion for Germany was 11%, with a further 35% being observed to have problematic general health literacy.

One of the population groups with a particularly high risk for low health literacy is unemployed persons, particularly the long-term unemployed, who have been unemployed for at least 1 year. Being unemployed is associated with poorer health outcomes such as increased risk of heart disease, mental illness, and lower physical health (8, 13–15), which in turn can lead to long-term unemployment. Although the unemployment rate in the European Union (EU) has been steadily decreasing since 2013 (from 10.8% in 2013 to 6.7% in 2019), the proportion for 2019 corresponds to more than 14 million unemployed persons aged 15–74 years (16). Quite a high proportion of these (41.8%) were long-term unemployed, and the proportion of long-term unemployed persons among those aged 55–74 years was almost 58%. In Germany, 898 000 persons were long-term unemployed in October 2020, almost a third of those registered as being unemployed (17). Most were aged 45 years and older (53%), did not have a vocational qualification (58%) and were male (56%). Slightly more than a quarter (26%) did not have German nationality.

While long-term unemployed persons have been shown to be at increased risk for poor health outcomes, the role health literacy plays in this regard and the respective needs of this population group are not clear. Survey data (8, 18, 19) suggest low levels of health literacy in this population. However, it is not clear whether members of this population group perceive themselves as having low health literacy, or indeed as having health information deficits or health literacy needs in general, as survey results are not necessarily communicated to or discussed with them. Further, findings of health literacy surveys do not automatically indicate the health topics that are relevant to those identified as having low health literacy scores. Health promotion activities involving unemployed persons have mainly focused on mental health and aimed to facilitate reintegration into the workforce (20). Exploring the subjectively perceived health literacy needs in these populations can serve as an entry point for participatory intervention development. Experts in the field have highlighted the importance of engaging directly with the members of the population group of interest when designing a health literacy intervention (3). The aim of this study was hence twofold: to systematically assess the current state of research with regards to health literacy needs of unemployed adults via a scoping review, and to empirically assess health literacy needs in a group of long-term unemployed adults via in-depth semi-structured qualitative interviews. Lastly, the findings of both approaches were integrated through triangulation.

## Methods

This study was conducted within the framework of a larger study on health literacy in unemployed persons (FORESIGHT; funded by the German Ministry for Education and Research). Using a parallel approach, we conducted a scoping review to obtain an overview of health literacy needs of unemployed persons identified and/or addressed in previous studies and also conducted in-depth semi-structured interviews with long-term unemployed persons participating in workforce reintegration programs to assess their health literacy needs. In line with the parallel data analysis approach (21), the collection and analysis of the scoping review and interview data was done separately.

The study was approved by the Ethics Committee of the University of Bremen, Germany (reference number 2020-26). Participation in the interviews was voluntary and all participants provided informed written consent.

### Scoping Review

We conducted the scoping review in line with the PRISMA-ScR guidelines for scoping reviews (22). The respective protocol was registered at the Centre for Open Science (OSF) (23) and is also provided as Supplementary Data (Supplementary File 1). Based on the PCC (Population, Concept and Context) criteria recommended for scoping reviews, we searched for primary studies with any designs that had been published in peer-reviewed journals or other sources (e.g., project reports, organizational reports, dissertations/theses). The studies had to have included persons officially registered as unemployed, looking for employment or participating in programs aimed at reintegration into the workforce, and assessed their health information needs, their health-related knowledge gaps, or components of health literacy that have been observed to be low in this population group. No date or language limitations were set at the search stage.

At the screening stage, non-primary studies such as literature reviews, editorials and conference abstracts were excluded from the scoping review, as were studies conducted in clinical settings or with clinical samples. Further, only studies published in English or German were included.

#### Information Sources and Literature Search

The following databases were searched for potentially relevant publications from inception to January 2021: MEDLINE via OvidSP, CINAHL via EBSCO, PsycINFO via EBSCO, Social Sciences Citation Index (SSCI) via Clarivate, Sociological Abstracts via ProQuest, and Applied Social Sciences Index and Abstracts (ASSIA) via ProQuest. The search terms, developed iteratively by the research team including a professional librarian include descriptors of unemployment, such as “jobless” or “laid off”, combined with descriptors of health literacy, such as “health knowledge” or “health promotion”. The MEDLINE search strategy is provided as Supplementary File 2. The other search strategies can be provided by the first author upon request.

To identify gray literature relevant to this review, two team members (JK and ML) independently searched websites of relevant national and international public health institutions (e.g., Kooperationsverbund gesundheitliche Chancengleichheit (Germany), Public Health England and Centre for Diseases Control and Prevention). They compared their findings and discussed these with the larger team.

#### Screening Process

Two authors, FSZ and HS, screened the titles and abstracts and then the full texts of the studies included into the next stage independently. Ensuing discrepancies were discussed by the two authors until consensus was reached.

#### Data Items and Data Charting Process

A data charting form was developed *a priori* and the team calibrated, tested and refined the draft before two team members (HS and JK) charted the data independently. Discrepancies that arose were resolved through discussion. Data items that were charted included study characteristics such as first author, year and type of publication, study design, definition of study population and sample size.

#### Synthesis of Results

The study characteristics, health literacy-related needs reported, and the methods used to assess these were narratively summarized.

### Qualitative Interviews

The qualitative part of the study was conducted in line with the Consolidated Criteria for Reporting Qualitative Research recommendations (COREQ, Supplementary File 3) (24). The research team characteristics are presented in Supplementary File 4.

At study onset, the project was presented to participants of a workforce reintegration program run in Bremen, Germany, by a partner organization of the FORESIGHT project. The organization offers different services such as recycling centers and second-hand furniture shops, where long-term unemployed persons take part in workforce reintegration programs. On average, the organization has 100 program participants at any given time. The information session took place during normal operating times and was also attended by the organization's social worker. The workforce reintegration programs are run such that the participants attend on a regular basis, for example, 4–6 h every weekday. For the participants, participation in the programs hence constitutes ‘going to work'.

#### Recruitment of Participants

Following the information session, the social worker, who was fully informed about the project from its conception, disseminated information about the study within the organization and invited program participants to take part in the interviews. No criteria were set for the recruitment of participants. For pragmatic reasons we decided on a sample of 10 persons. The interviews were conducted between January and February 2021. The interviewers (FSZ and HS) did not know any of the interviewees prior to the study.

#### Interview Guide

In-depth semi-structured interviews were conducted using a guideline developed in consultation with the social worker at the partner organization (see Supplementary File 5 for the original version and the English translation) and focused on themes such as the health topics of interest to the participants, the health services and sources of health information they use, and the barriers/facilitators they experience when accessing health services or health information.

The interviews were conducted at the partner organization, in a closed room in which only the interviewer and interviewee were present, and during normal operating hours. The interviewees were offered an incentive of 50 Euro for their participation and were interviewed once. Before the interviews, participants provided written informed consent. All interviews were conducted in German and were audio-recorded and later transcribed verbatim in the original language of the interviews. The duration of the interviews varied from 8 min to almost an hour. Transcript segments required for this manuscript were translated into English by FSZ and ML and TB cross-checked the translations.

#### Data Analysis

The interview transcripts were analyzed using thematic analysis via the freely available online program, QCAmap (25). The program foresees the compilation of questions to be addressed during the analysis. To this end, one of the authors (FSZ) initially compiled a list of such questions based on the interview guide. She then read through two of the interviews, using the list to code the respective segments and adding further questions where applicable. Thereafter, two coders (FSZ and ML) independently pilot coded two interviews each by assigning “categories” (codes) to the relevant interview segments inductively. The coders compared their results and addressed any disagreements that arose. They then used the developed category structure to code the rest of the interviews, again independently. Further questions and categories were added as deemed necessary (see Supplementary File 6 for final list of questions used to analyze the data). After all interviews had been coded, the two coders went through all the interview transcripts, comparing their coding. They discussed any differences until consensus was reached. An example of the coding frame used to code one of the questions is provided as Supplementary File 7.

In a second step, the analysis questions and corresponding transcript segments were classified according to factors relating to different components of health literacy: finding health-related information, understanding, appraising and applying health-related information. Potential barriers as well as resources available to participants for each of the health literacy components were then identified based on the responses to the different analysis questions.

## Results

### Scoping Review

**Figure 1:**
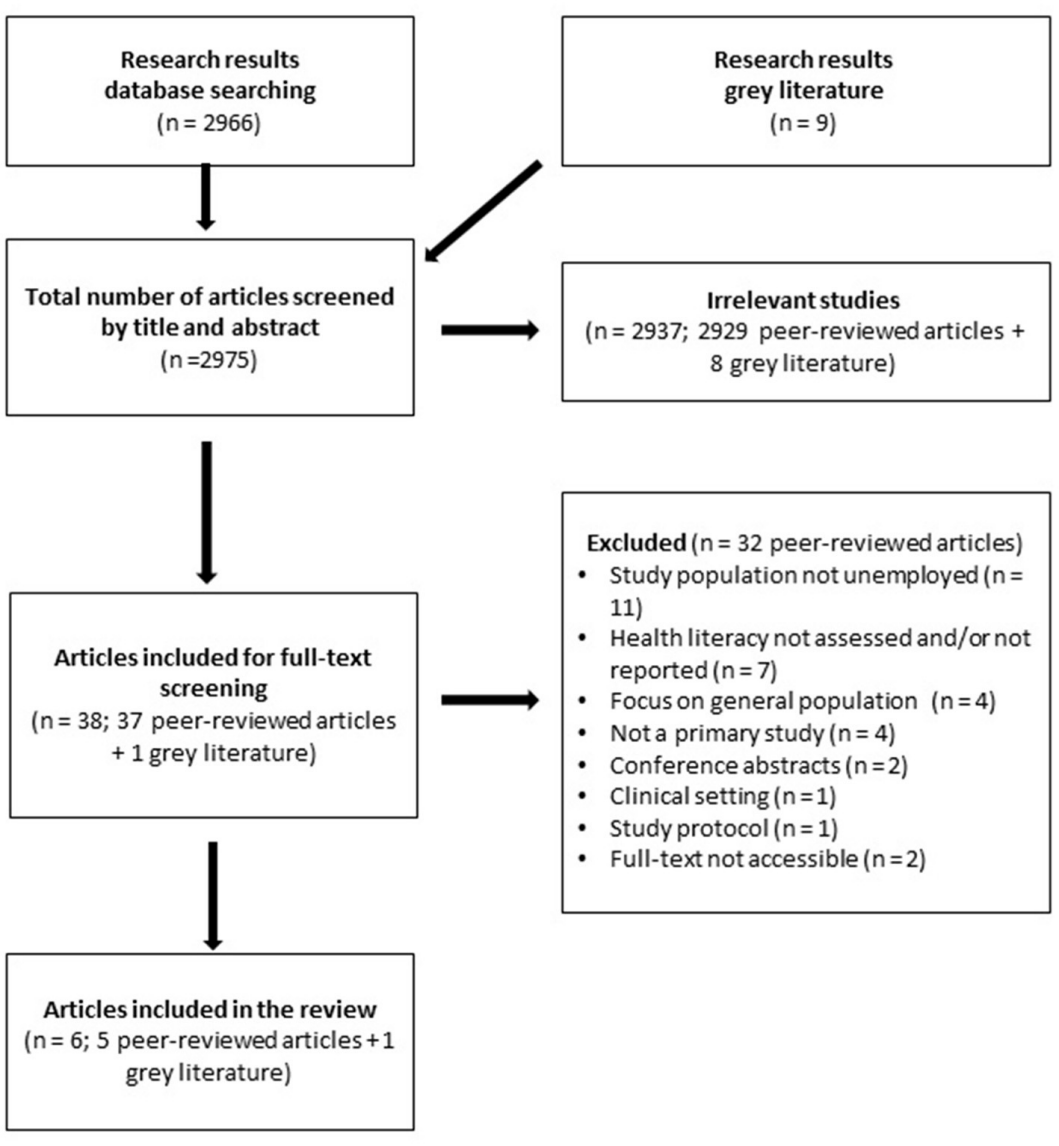
PRISMA flowchart showing selection process of publications included in the review.

From 2966 titles and abstracts of peer-reviewed articles that were screened, 36 were included in the full-text stage, and five were included in the final review. The main reasons for exclusion were (a) wrong study population (focus not on unemployed adults), (b) health literacy not assessed or reported and, (c) not primary data. Details are described in the PRISMA flow chart (Figure 1). The list of excluded full-texts is provided as Supplementary File 8.

Nine potential publications were identified through the gray literature search, seven of which were reports, one manual and one preprint manuscript. Only one of the reports was included in the review.

#### Place of Study, Study Characteristics, and Outcomes Assessed

Two of the included peer-reviewed articles were based on studies conducted in Finland (26) and Portugal (27). The other three (28–30) and the report (31) were based on studies conducted in Germany. None of the studies identified included participants of a workforce-reintegration program.

A summary of the data extracted for each of the articles and the report is presented in Table 1. The full data is provided as Supplementary File 9. The study conducted in Finland investigated the relation between information seeking practices and coping strategies among 750 long-term unemployed persons, focusing on everyday life information and health information. Coping was assessed using abstracts from Folkman and Lazarus' revised Ways of Coping questionnaire and use of information was assessed using open questions.

**Table 1:** Characteristics and summary of findings of the five peer-reviewed and one gray literature source (31) included in the scoping review.

**References, country**	**Study design/aims/outcomes**	**Participant characteristics**	**Health Literacy (HL) measurement/needs assessment**	**Findings**
Pertillä et al. (26). Finland	•Cross-sectional survey •Aims: Investigate information seeking practices and coping strategies of long-term unemployed via questionnaire; study link between coping functions and everyday life information seeking. •Outcomes: information seeking behavior, coping strategies for unemployment	•750 long-term unemployed persons, •recruited via Ministry of labor •73% >54 years	•Participants asked how often they sought information about health on scale from 1 (try to avoid such information) to 6 (very often) •Questionnaires used to determine needs regarding information seeking relating to unemployment and health	•High mix-focused copers most active information seekers concerning both unemployment and health •Coping functions linked to information seeking practices of participants •High problem-focused copers significantly more active in information seeking than medium and low problem-focused copers •Mixed-focused copers most active regarding problem-specific information seeking
Santos et al. (27). Portugal	•Delphi technique (2 rounds) •Aims: Create expert consensus regarding how to develop and implement an intervention program for mental health promotion among unemployed people •Outcomes: Consensual items for mental health intervention for unemployed	•46 experts (mental health; employment; temporary work; psychiatric disorders prevention) •recruited via snowball sampling •mean age: 48,17+-12.48 years	•Mental Health Literacy (MHL) defined as identifying signs and symptoms of depression, anxiety and stigma regarding mental health •Importance of contents and skills to be promoted by intervention rated via 5-point Likert scale (1= totally disagree and 5 totally agree)	Important intervention components identified: •promotion of MHL (regarding anxiety, mood disorders and stigma) •methods to challenge unemployment (promotion of job searching skills through job-interviewing training) •mental health promotion skills (self- regulation of emotions, effective communication training, awareness of skills and personal facets) •preferred structure: small groups (up to 10 participants) on more than 10 weekly sessions (each 2 h) Main outcomes to be measured: •participants' satisfaction with intervention •indicators of mental health (as anxiety or general psychosocial functioning)
Staiger et al. (28). Germany	•Semi-structured interviews •Aims: Identify barriers to and facilitators of help-seeking and service use based on experiences of unemployed people with mental health problems •Outcomes: Experience with help-seeking and mental health service use with a focus on barriers and facilitators	•15 (7 female / 8 male) unemployed persons with self-reported psychological distress •recruited via employment agencies and social organizations •aged 19-63 years (mean 48) •unemployed for 2 months to 15 years	•HL assessed as knowledge-related facilitators and barriers of service use, e.g. What do you know about mental health and its prevention? If you had a mental illness where would you seek help? •Experiences regarding stigma and discrimination, and needs concerning structures and conditions of health care also assessed	Main barriers of help-seeking: •fear of side effects of psychopharmacological treatment (low MHL) •ineffective psychiatric help •perceived discrimination by mental health care professionals •stigma in the social environment •GP's lack of interest in mental health problems Main Facilitators of help-seeking: •gaining knowledge as motivation factor for treatment •awareness and acceptance of illness •GP as facilitator and supporter •positive relationship between patient and therapist
Waldmann et al. (29). Germany	•Cross-sectional survey •Aims: Investigate the influence of MHL on help-seeking intentions and behaviors in unemployed people with mental health issues using questionnaire •Outcomes: MHL, depression-related knowledge and attitudes toward treatment and treatment options	•301 unemployed persons with mental health problems (50.2% female) •mean age 43.7 years •recruited via employment agencies •average unemployment time 35.5 months	•MHL assessed using Mental Health Knowledge Schedule (MAKS), Depression Literacy Scale (DLS) and Depression with Suicidal Thoughts Vignette. •Help-seeking intentions and behaviors assessed using •General Help-Seeking Questionnaire (GHSQ)	•Higher MHL associated with increased help-seeking intentions and behaviors (from health professionals and from family and friends) •Age negatively associated with intentions to seek help from family and friends, while female gender positively associated •Having symptoms positively associated with seeking help from professionals but negatively associated with seeking help from family and friends.
Wigand et al. (30). Germany	•Longitudinal study •Aims: Assess predictors of help-seeking among unemployed people with mental health problems •Outcomes: Barriers and predictors of help- seeking, MHL, depressive symptoms, beginning of mental health treatment within 6 months after baseline survey	Baseline: •301 unemployed persons with mental health problems (50.2% female) •mean age 43.7 years •recruited via employment agencies •average unemployment time 35.5 months Follow up: •270 unemployed persons (50.7% female) •mean age 44 years •average unemployment time 36.4 months	•MHL assessed using the 8 treatment- related items of the 22-item DLS •Depressive symptoms were measured using the Patient Health Questionnaire •Frequency of symptoms assessed over the last 2 weeks (from ‘not at all'/0 to ‘nearly every day'/3): e.g., feeling tired or having little energy/interest/ pleasure in doing things	•Following factors significantly predicted new help-seeking during follow-up period in different models: •female gender (Odds Ratio (OR): 1.82; 95% Confidence Interval (CI): 0.97-1.02) •more depressive symptoms (OR: 1.08, 95% CI: 1.02-1.14) •higher MHL (OR: 1.22; 95% CI: 1.03-1.46) •fewer non-stigma-related barriers (OR: 0.28; 95% CI: 0.12- 0.63) •mental health service use at baseline (OR: 3.44; 95% CI: 1.57-7.57)
Wieland and Hammes (31). Germany	•Cross-sectional survey •Aims: Explore HL and the abilities of German citizens to cope with illnesses using online questionnaire (question part of a larger health report) •Outcomes: HL, psychological health type, health knowledge, health behavior	•1417 unemployed people (from total of 4764 participants, all BARMER GEK health insurance company members) •mean age 61.3 years •58.8% women	•HL determined via 10 different questions developed by Wieland & Hammes (32). All questions ranked on a scale from 0 (it's not the case at all), to 4 (it's very often the case)	•unemployed had significantly higher HL compared to employed (2.61 vs.2.53), but reported lower health status and health knowledge than employed persons •unemployed spent significantly more time weekly on health-related activities (2.84 vs. 2.47 h) •no difference observed between unemployed and employed persons regarding association between HL and health factors such as nutrition, physical activity, stress management and family/partnership, however, unemployed ascribed less relevance to the stated factors, except for physical activity. In particular stress management was accorded little relevance. •Participants with lower HL also spent less time on health-related activities

The study population for the Portugal study comprised 46 experts from various fields with professional experience in mental health, employment/temporary work and prevention of psychiatric disorders (27). The study applied the Delphi technique to reach expert consensus regarding essential intervention components for a program to promote mental health among unemployed people.

Two of the three articles from Germany (29, 30) were based on the same study population comprising unemployed persons with mental health problems who were recruited via unemployment agencies. One of the articles investigated the influence of mental health literacy (MHL) on help-seeking intentions and behaviors of the participants (29), while the other investigated predictors of help-seeking among the participants (30). The outcomes were assessed using the Depression Literacy Scale (DLS) (29, 30) and the Mental Health Knowledge Schedule (MAKS) and the Depression with Suicidal Thoughts Vignette (29). The third article from Germany assessed barriers and facilitators of help-seeking and service use among 15 unemployed persons using in-depth interviews (28).

The report summarized results of an online survey to assess health literacy conducted among 4,764 members of the Barmer GEK health insurance company, 29.7% of whom were unemployed (31). Health literacy was measured using a self-developed questionnaire closely oriented to the professional self-efficacy scale.

### Summary of Findings of Studies Included in the Scoping Review

#### Information Seeking and Health Literacy Needs of Unemployed People

In the study by Perttilä and colleagues (26), the participants generally tended to seek information about health more often than about unemployment. In general, health-related information seeking was more prevalent among high and medium copers compared to low copers. Looking at coping strategies, health-information seeking was highest among high copers using a combination of emotion and problem-focused strategies compared to those using either emotion-focused or problem-focused strategies.

In the Barmer GEK survey (31), the average health literacy score among the unemployed was slightly higher than that for those employed. The data suggests that neither age nor gender differences explained this difference. The results of the survey indicate that expectations concerning success in staying healthy received the lowest score and may therefore be regarded as an area of need for an intervention.

#### Mental Health Needs of Unemployed People

From the data synthesis, two major topics were identified regarding mental health needs of unemployed people, namely (a) potentially important intervention components for unemployed people and (b) facilitators and barriers of help-seeking among unemployed people with mental health problems.

The first topic was identified in the study by Santos and colleagues (27), with the participating psychologists and psychiatrists agreeing that the following aspects comprised important intervention components: promotion of MHL (mainly about anxiety, mood disorders and stigma about mental health), methods to challenge unemployment (as promotion of job searching skills through job interviewing training) as well as mental health promotion skills (self-regulation of emotions, effective communication training, awareness of skills and personal facets). Regarding the structure of the interventions, the experts recommended that these be conducted with small groups (up to 10 participants) and comprise more than 10 weekly 2-h sessions.

The studies conducted in Germany identified the second topic: barriers as well as facilitators of help-seeking among unemployed people with mental health problems. The barriers identified included: the fear of side effects of psychopharmacological treatment (rated as low MHL), ineffective psychiatric help, perceived discrimination by mental health care professionals, stigma in the social environment and general practitioners' (GPs) lack of interest in mental health problems. Facilitators of help-seeking identified were: gaining knowledge as motivation factor for treatment, awareness and acceptance of the illness, GP as facilitator and positive relationship between patient and therapist (28). Additionally, factors such as female gender, higher MHL, more depressive symptoms and more self-identification as having a mental illness significantly predicted increased help-seeking intentions (29).

### In-depth Semi-structured Interviews

Seven men and three women aged between 30 and 58 years took part in the interviews. Six of the interviewees were older than 50 years and three were younger than 40 years. Their duration of unemployment ranged from 5 to 19 years, and four of them had been unemployed for more than 15 years. All but one were native Germans.

The findings of the interviews are summarized hereafter according to the different health literacy aspects. The original versions of the quotes used are provided as Supplementary File 10.

#### Finding Health-Related Information

The participants reported getting health-related information from various sources, including official health service providers such as the general physician, health insurance company and pharmacist, as well as digital and print media (Table 2). Seven of the ten participants also mentioned the social worker at the organization as a source of health information.

**Table 2:** Sources of health information used as well as preferred by the qualitative interview participants.

		**Non-digital**				
	**Internet/**	**Official health**	**Workplace**	**Family and**	**TV/news/**	
	**digital media**	**service provider**		**friends**	**magazines**	**Flyer, face-to-face**
Interview 1						
Interview 2						
Interview 3						
Interview 4						
Interview 5						
Interview 6						
Interview 7						
Interview 8						
Interview 9						
Interview 10						

*
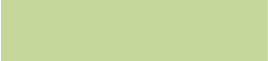
 Used sources of information.*

*
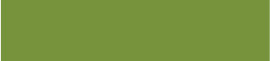
 Used and preferred sources of information.*

Nine out of 10 participants reported using the Internet as a source of health information, and five stated the Internet as their source of preference. Among those who preferred the Internet, the easy availability of information was the main reason given, especially in comparison to print media.

**Figure 2:**
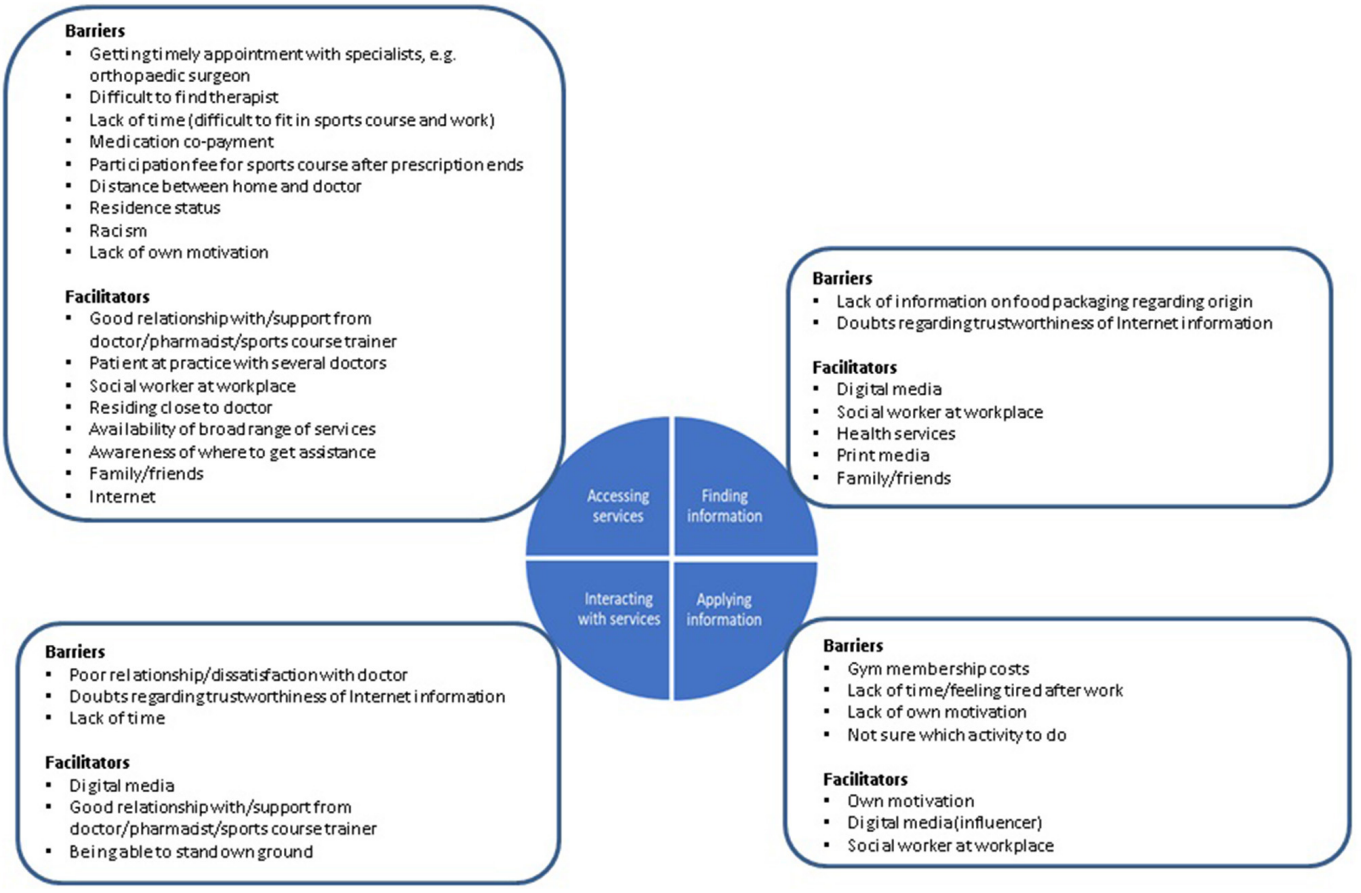
Barriers and facilitators regarding the health literacy components identified from interview data.

The participants generally reported searching the Internet for health information when requiring specific information. For instance, one participant reported how she had searched the Internet to try and understand more about colonoscopy after having been referred for the procedure. Another reported that his partner suffered from panic attacks and how information from the Internet had helped him realize that the condition does exist and also to understand it better. In general, digital media, together with the social worker at the “place of work”, health services, as well as family and friends were reported to facilitate access to health-related information (Figure 2).

The participants reported hardly any barriers related to finding health-related information. One participant reported not being able to find information about the origin of dairy food products on the packaging, while those who reported using health information from the Internet generally doubted the trustworthiness of the information. According to one participant:

*But the problem of the Internet is also; there I always have a but, because sometimes they also exaggerate. You read sometimes, for example: I have this, and I know how I feel, then I write that down there. The next person has the same, but describes it differently, because he feels differently. That's also the question: what to believe?* (Interview 9, male, 39 years)

The extent to which digital media was used as well as the purposes varied among the participants. For instance, one participant for whom nutrition and physical activity played a very important role followed advice from “influencers” on YouTube and Facebook.

*Well, to be honest, I admit that I watch a lot on the Internet and YouTube because of my wife. Because there is not only something about nutrition, but there are also all these people… influencers. Of course, you don't believe some of them because they want to sell their stuff, but I think some things are also true. They show you how to lose weight if you're overweight, what to eat, how many calories to eat and what not to eat. So I kind of take a lot from the Internet: YouTube, Instagram and Facebook*. (Interview 9, male, 39 years)

According to the participant, he however only “followed” people whose body image represented what he deemed to be physically fit.

*I look at these people more because of nutrition, to do sports. Because they explain to you that, if you want to train your abdominal muscles, for example, you first have to eat this and that, and train this for the exact goal. Or if you want to develop your chest muscles (….). Well, always these athletes, not only…. I can't look at someone who looks like this [*indicates an overweight person using arms*] and explains to me about nutrition. Of course not*. (Interview 9, male, 39 years)

#### Accessing Healthcare

All participants had health insurance coverage and hence did not have any formal difficulties accessing healthcare. Whereas some of the barriers mentioned pertained to individual participants, for example, medication co-payment, residential status and racism, aspects such as difficulties getting a timely appointment with a specialist, problems finding a therapist and the distance between the home and the physician's practice were mentioned by several participants.

When it comes to accessing healthcare, having a good relationship with one's doctor and having his/her support were generally reported to facilitate access to healthcare. A good relationship with the doctor was often linked with trust and feeling well-taken care of. In some cases, the doctor assisted with getting a timely appointment with a specialist.

The social worker at the “place of work” was also reported to facilitate access to healthcare, either by giving advice regarding where to go for help or assisting with the filling out of application forms for services required. One of the participants described this as follows:

*I mainly talk to Mrs. S [*the social worker, for advice*], because I think she is the one who also applies for [*new orthopedic working shoes*] or helps check what is possible*. (Interview 6, female, 30 years)

Referring to a time when she was not feeling well psychologically, the same participant went on to say:

*I talked to Mrs. S [*the social worker*] and tried to find* [with her] *a therapist again, because it would be better if I had one*.

Other facilitators mentioned by the participants were the Internet, family and friends, as well as being a patient at a practice with more than one doctor (Figure 2). Examples of statements made by the participants regarding how the Internet facilitates access to health information are:

*Well, I guess the easiest way… because even with some doctors it's currently… is the Internet. I can say the Internet. Exactly. Although they are not doctors, but on the Internet, one really has a lot of information there. Whether one really believes it or not, but the information is there*. (Interview 9, male, 39 years)

#### Use of /Interaction With Healthcare Services

A good relationship and support from the healthcare provider were not only reported to facilitate access to healthcare, but also use of services, respectively positive interaction with the healthcare provider (Figure 2). Participants with a good relationship to their healthcare provider generally reported being satisfied with the interaction and services they received. While most of the participants referred to the doctor in this respect, one mentioned the pharmacist.

*I'd rather ask the doctor or the pharmacist. And that's also a decent man* (The Pharmacist), *he also takes a lot of time for the people, that's good (...). Really, not just prescription, out, in, goodbye and…, no, no, he still talks to the people. That's good. I think that is really cool*. (Interview 1, male, 53 years)

A further participant described how she had stopped attending a gymnastic course for her back because the trainer with whom she had a good relationship offered the course during her normal “working hours” and she could not get the time off. She had tried attending a course offered at a different time but did not feel as comfortable with the other trainer.

*The [*aqua fitness*] course with the trainer with whom I got along well was unfortunately during my “working hours”. That didn't work out so well with my working hours. (…)*

*I also once did the same course with another trainer (...), she was also quite friendly, that was also quite good, but somehow I didn't have the same connection to her (...), I somehow found it better with the other trainer*. (Interview 6, female, 30 years)

Dissatisfaction with the services received was mainly mentioned in relation to the participants feeling that the doctor was not paying them enough attention and was just dealing with them as if with numbers.

*At the doctor's (…) you go there, for example you can say “I have stomach ache today” and you get paracetamol. Tomorrow I go there and I say “I have a headache” and I get ibuprofen and paracetamol. They give you the same stuff, it's like that. Sometimes before I even go there I say, I'd rather go buy paracetamol. Because I know if I go, I'm going to get paracetamol*. (Interview 9, male, 39 years)

In most cases the dissatisfaction led to a change of doctors. One participant however reported how standing his own ground had helped him get the necessary treatment after consulting his doctor with longstanding throat pain.

*I was there 2 years ago, I had laryngitis. I knew that it was [*not*] a normal cough and he would prescribe me ACC Acute. I also told him that I don't need ACC and that it's been going on for a while. I can tell this is not normal. “Okay, then I'll give you a referral for the ENT specialist,” he said. And then I went and he said, yes, laryngitis and antibiotics. And I had told the doctor before “don't I need antibiotics or something?” (…) and he gave me ACC*. (Interview 7, male, 52 years)

Regarding preventive measures such as dental and medical check-ups, four of the 10 interviewees, all male, reported that they did not take part in any, not even the dental check-ups. The reasons given for not attending the latter were no time, childhood trauma and no need. The interviewee reporting no need said he had already lost almost all his teeth and was just waiting for one more to fall out, after which he would get dentures.

The other six interviewees reported at least going for dental check-ups, although one female interviewee did not do so annually, but rather now and again. All three female interviewees reported going for annual gynecological check-ups and two of them also went for general medical check-ups. Only one of the male interviewees reported participating in a further preventive measure, namely, back training offered by the social worker at the work reintegration organization.

#### Application of Health Information

The health information the interviewees mentioned in this regard mostly concerned nutrition and physical activity. Five of them specifically referred to the importance of both nutrition and physical activity, while a further three referred only to physical activity and one other only to nutrition. While some of the interviewees mentioned the social worker at the organization, digital media and personal motivation as facilitating factors (Figure 2), all of them reported difficulties when trying to put their knowledge into practice. A common barrier reported was time, respectively “work-related” difficulties, with some of the interviewees stating that their work was so physically demanding that they were too tired to prepare a healthy, balanced meal or do any physical activity after hours.

*During the week it's not so good, because in the evening I don't feel like cooking and here “at work”* [in the canteen] *there are hardly any vegetables*. (Interview 7, male, 52 years)

*I am exhausted* [after “work”] *because I also “work” physically. As already said, then there's something small to eat, not always healthy. When it has to be quick, it's a can* [of food], *but there is always an apple with it*. (Interview 10, male, 54 years)

The participant however also explained how he still tries to balance everything as follows:

*But I try somehow, as already said, to keep a well-balanced diet. The good thing here is that I have the exercise, so exercise and sports are actually always part of it, but I'm so busy here that during the weekend I somehow also... But I do have my quite good... quite well- balanced moments, where this eating, processing, the food, moving - without putting on weight - I'm diabetic. I am sometimes more or sometimes less disciplined. I know how it goes, I took part in a diabetic training course, I sometimes sin. But then again I have a day where I have to, I don't know, climb stairs a hundred times. I always try to balance things a bit and that works out quite well for me*. (Interview 10, male, 54 years)

Some interviewees also reported barriers specific to themselves, such as lack of own motivation:

*Well, theoretically everything is possible for me, but practically it is not so good. So, for example, at times I think I should actually be more physically active, but I don't do anything about it. That's the simple sentence…. Well, because I do know, because I'm not 20 anymore and with 20 one simply didn't know certain things and now with over 50 one does know more and that's why, the problem is just the implementation. I can't really answer why*. (Interview 5, female, 55 years)

A similar personal barrier reported was feeling down:

*I've tried* [to work] *a little bit on nutrition itself, or I always try a bit not to eat too much sugar or too much fatty stuff, but when I really notice that I'm not feeling so good mentally at the moment, then I eat whatever tastes good to me at that time. Be it the soggiest, greasiest burger or whatever*. (Interview 6, female, 30 years)

### Triangulation of Scoping Review and Qualitative Data

Throughout the interviews, most of the participants showed that they were quite knowledgeable about health topics such as nutrition and physical activity. The problem was rather putting the knowledge into practice. The main barrier mentioned in this regard was lack of motivation, particularly among those who lived alone. Several participants reported finding it difficult to find the energy to cook for themselves or do some physical activity alone.

**Figure 3:**
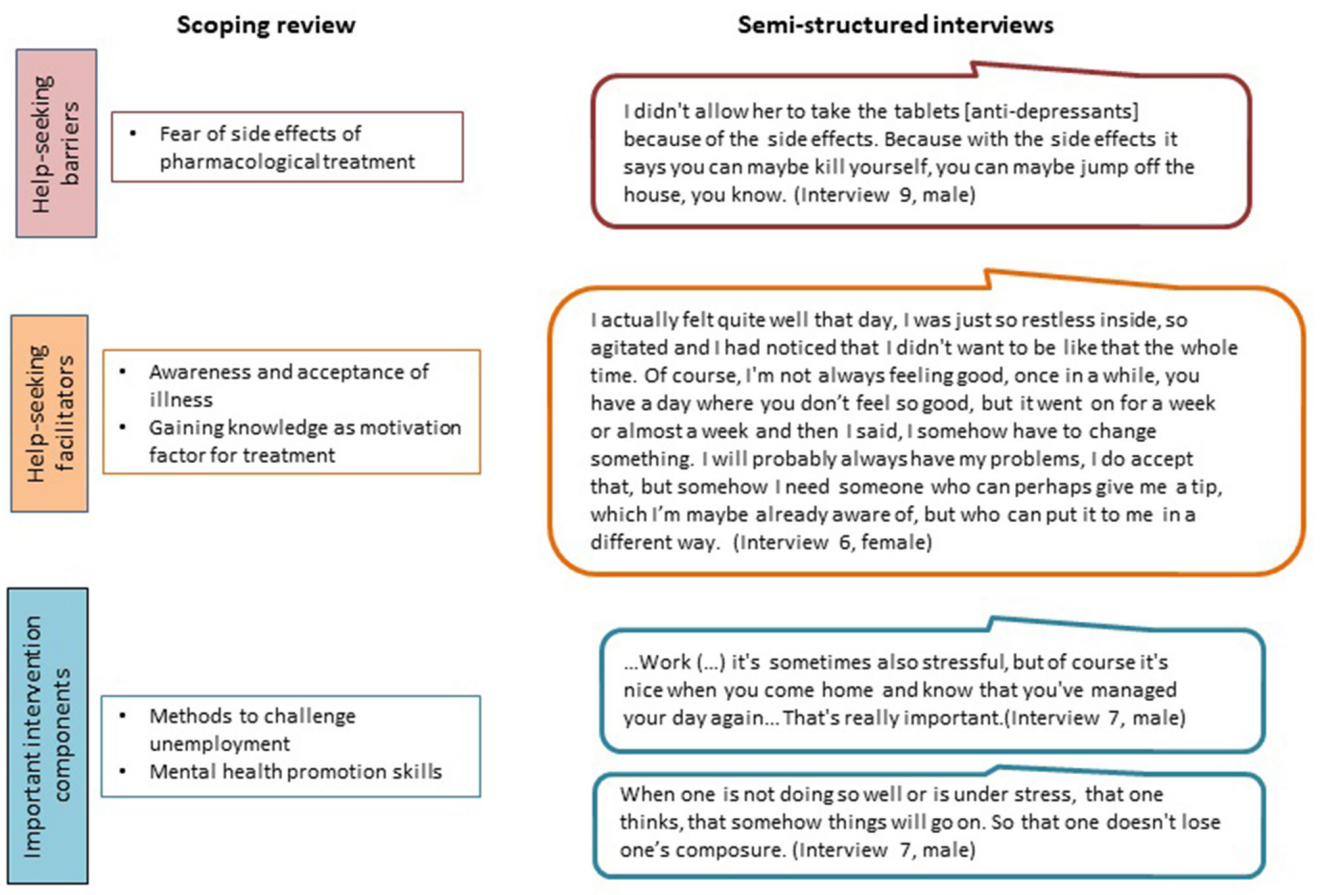
Triangulation of scoping review and interview data.

Although health was taken for granted by some participants, it was identified as one of the main sources of their quality of life. Regarding mental health, the results of the scoping review on some of the barriers and facilitators as well as intervention components identified as being important, resonated with some of the interview findings (Figure 3). This particularly concerned the fear regarding side-effects of antidepressants, the importance of being aware of one's condition and accepting it, and the significance of having a job, respectively something to do to help structure the day. The need for mental health promotion skills was also identified during the interviews, with the participants mentioning active relaxation and avoiding stress as some of the topics of interest to them, in addition to nutrition and physical activity, among others.

Concerning facilitators of help-seeking when having mental health problems, health professionals such as the GP and therapist, and family and friends were identified as playing a role in both instances of the analysis.

## Discussion

In this study, results from a scoping review and in-depth semi-structured interviews were triangulated to assess the health literacy needs of long-term unemployed persons. The triangulation process highlighted similarities between barriers and facilitating factors across these data sources. Although unemployment is generally associated with poor health and low health literacy (8, 13, 18, 19), our study participants were well-informed about health topics and relevant information sources, partly because of their own history of illnesses. Thus, finding and understanding health information was not a major issue among our participants. Rather, we identified applying health information, i.e., developing or maintaining a healthy routine in everyday life, as the main problem.

While large parts of the general population probably face similar challenges regarding engaging in healthy lifestyles, the situation for long-term unemployed persons is compounded by various factors such as low economic resources and limited supportive social networks (33, 34). Our findings indicate that unemployed persons do not necessarily show low levels of health literacy, which is in line with the results of the report included in the scoping review, whereby unemployed persons on average had slightly higher health literacy scores compared to those employed (31). Recent results of a cross-sectional survey conducted in Austria also contradict the common assumption regarding employment and health literacy. In this study (35), the authors assessed general health literacy among adults residing in a disadvantaged district with high cultural and ethnic diversity as well as a considerably high unemployment rate, and compared it to adults residing in Vienna and to the general Austrian population. Health literacy was observed to be highest among participants from the disadvantaged district.

On the whole, most of the barriers and facilitators reported by our study participants concerning finding and applying health information, as well as accessing and interacting with services, correspond to those found in the population at large. As has been observed in other studies, having access to the Internet/digital media facilitated the finding of health-related information at the individual level (36–39), although some skepticism was also raised regarding the trustworthiness of online information.

### Practical Implications

This study was conducted as a first step in an intervention development process. There are several practical implications that can be derived from our analysis. While the identified literature mainly focused on mental health literacy, our study participants also highlighted healthy nutrition and physical activity as relevant topics. When designing intervention components, the main focus should be placed on applying health information in terms of developing healthy routines. The reintegration program seems to be a good place for promoting health literacy for a number of reasons. Firstly, the social worker at the organization appeared to be a relevant source of health information and may also provide cues to action to potential intervention participants. Further, intervention delivery at the organization would not only lower the threshold for taking part, the participants themselves could also be involved in the development and delivery of the intervention, for example as local champions for certain topics. Such participatory formats could also help to overcome motivational barriers and might increase participants' sense of having control over their own lives (6, 40, 41).

### Strengths and Limitations

The main strength of this study is the integration of findings from the literature and from qualitative interviews with long-term unemployed persons. Regarding the latter, taking an open approach, whereby study participants were asked for health topics of interest to them and then identifying their health literacy needs from the interview data ensured that the perspective of the population group of interest was represented. The participatory approach further helped provide insight into barriers and facilitators as well as topics of interest to the study population that research has hardly focused on. A subsequent intervention development workshop with long-term unemployed persons will build on the insights gained, further supporting co-creation.

The fact that the interviews were conducted with participants of a workforce-reintegration program in Germany limits the generalizability of the findings to long-term unemployed persons in general, or in other countries, in particular those without workforce-reintegration programs. Our study population benefited from health-related activities offered as part of the reintegration program, which could have led to their relatively high levels of health literacy.

Another limitation is that all our participants were German-speaking. Long-term unemployed persons not able to speak or communicate sufficiently in German might face other difficulties dealing with health-related information or interacting with healthcare services.

The small number of articles identified by the scoping review limited the triangulation of the scoping review and qualitative data, especially as the identified literature mostly focused on mental health literacy. Further, the identified studies, including the gray literature, were all from Europe and did not include workforce-reintegration participants. This last aspect has possible implications regarding identification of barriers to application of health information. Our qualitative study identified barriers related to participating in the reintegration program, such as being tired or not having enough time, which were not identified by the scoping review. On the other hand, unemployed persons without access to reintegration programs may have more difficulties in finding and appraising health information. In addition, the three articles on mental health literacy with primary data were all from Germany, with two of them being based on the same study population. Nevertheless, as has already been discussed, our findings regarding barriers and facilitators are in line with the literature.

## Conclusion

Our results highlight a challenge to population-based health literacy interventions, that is, the need for interventions that not only aim to improve health literacy scores, but also help translate health literacy scores into practice. In countries where long-term unemployed persons are engaged in official job reintegration programs, the organizations running such programs can serve as low threshold intervention sites, with the unemployed themselves playing a central role in the design of the interventions.

## Data Availability Statement

The datasets presented in this article are not readily available because the informed consent signed by the participants did not include their agreeing to their qualitative data being shared publicly. Requests to access the datasets should be directed to Tilman Brand, brand@leibniz-bips.de.

## Ethics Statement

The studies involving human participants were reviewed and approved by the Ethics Committee, University of Bremen (reference number 2020–26). The patients/participants provided their written informed consent to participate in this study.

## Author Contributions

BS, TB, and HZ conceptualized the study. HS, ML, JK, LC, and FS-Z were involved in the process of conducting the scoping review. FS-Z, TB, BS, HZ, ML, JK, and HS were involved in developing the qualitative study. FS-Z and HS conducted the interviews, which were coded and analyzed by FS-Z and ML, together with TB. FS-Z drafted the manuscript. All authors were involved in writing up the protocol of the scoping review, revised it critically, and approved the final version.

## Funding

This research was funded by the Federal Ministry of Health, Germany, GRANT ZMVI1-2519FSB020. The publication of this article was funded by the University of Bremen Library - Staats- und Universitätsbibliothek Bremen.

## Conflict of Interest

The authors declare that the research was conducted in the absence of any commercial or financial relationships that could be construed as a potential conflict of interest.

## Publisher's Note

All claims expressed in this article are solely those of the authors and do not necessarily represent those of their affiliated organizations, or those of the publisher, the editors and the reviewers. Any product that may be evaluated in this article, or claim that may be made by its manufacturer, is not guaranteed or endorsed by the publisher.
