# Supplementary material for: Health Literacy Needs Among Unemployed Persons: Collating Evidence Through Triangulation of Interview and Scoping Review Data
Source: Front Public Health. 2022 Feb 22;10:798797. doi: 10.3389/fpubh.2022.798797 (PMC8902044; doi:10.3389/fpubh.2022.798797)
Supplement: Supplementary file 1 [file Data_Sheet_1.ZIP › Supplementary file 1_Scoping review protocol.pdf]

Supplementary file 1: Scoping review protocol

Health literacy needs among unemployed persons: collating evidence through triangulation of interview and scoping review data

Authors:

Florence Samkange-Zeeb[(1)], Hunny Singh [(2)], Meret Lakeberg [(1,2)], Jonathan Kolschen [(2)], Benjamin Schüz[(2)], Lara Christianson[(1)], Karina Karolina De Santis[(1)], Tilman Brand[(1)], Hajo Zeeb[(1,2)]

(1) Leibniz Institute for Prevention Research and Epidemiology – BIPS. Department of Prevention and Evaluation

(2) University of Bremen, Faculty of Human and Health Sciences (Public Health)

Corresponding author: Hajo Zeeb, zeeb@leibniz-bips.de, Tel: +49 421 21856902

Table of contents

General information 3

1. Title 3
2. Research team and organisational affiliations..... 3
3. Timeline 3
4. Funding sources 3

Project details according to the PRISMA-ScR checklist 4

Item 1. Title 4

Item 2. Abstract 4

Item 3. Rationale 4

Item 4. Objectives 4

Item 5. Protocol and registration..... 5

Item 6. Eligibility criteria 5

Item 7. Information sources 6

Item 8. Search 6

Item 9. Selection of sources of evidence 6

Item 10. Data charting process 7

Item 11. Data items..... 7

Item 12. Critical appraisal of individual sources of evidence 7

Item 13. Summary measures 8
Item 14. Synthesis of results 8
Item 15. Risk of bias across studies 8
Item 16. Additional analyses 8
Items 17-26: Results / Discussion 8
Item 27: Funding 8
Appendix: PRISMA extension for scoping reviews (PRISMA-ScR) checklist 9

General information

1. Title

What are the health literacy needs of the unemployed? A scoping review

2. Research team and organisational affiliations

1. Prof. Dr. Hajo Zeeb [1,2]
2. Dr. Florence Samkange-Zeeb [2]
3. Prof. Dr. Benjamin Schüz [1]
4. Dr. Tilman Brand [2]
5. Dr. Karina De Santis [2]
6. Hunny Singh [1]
7. Meret Lakeberg [1,2]
8. Jonathan Kolschen [1]
9. Lara Christianson [2]

Organisational affiliations:

(1) University of Bremen, Faculty of Human and Health Sciences (Public Health)

(2) Leibniz Institute for Prevention Research and Epidemiology – BIPS, Department Prevention and Evaluation

3. Timeline

1. Start date: 01.10.2020
2. Anticipated completion date: 30.09.2021
3. Project stage at registration (01.02.2021):
    - Preliminary searches conducted in January 2021
    - Study selection and data coding not yet initiated

4. Funding sources

German Federal Ministry of Health, grant number: ID ZMVI1-2519FSB020

Project details according to the PRISMA-ScR checklist

Item 1. Title

What are the health literacy needs of the unemployed? A scoping review protocol

Item 2. Abstract

Not applicable at the protocol stage

Item 3. Rationale

A considerable proportion of the general population worldwide has limited health literacy, that is to say, has difficulties to find, understand and use health information and services they require to make appropriate health decisions. Low health literacy is more predominant among those with less financial resources, low level of education, low socioeconomic status or older age. Because low level of education increases the risk of unemployment, it can be assumed that unemployment is associated with low health literacy. However, little is known about health literacy needs in this population. The intervention studies involving the unemployed typically focus on measures to improve their mental health and thus to facilitate their reintegration into workforce. The present study aims to investigate the health literacy needs in the unemployed using a scoping review of the literature. We aim to map the methods used to assess such health literacy needs, including how the participants were recruited and how the topic focused on was determined.

Item 4. Objectives

This scoping review is being conducted within the context of a project that aims to obtain an overview of the needs of the long-term unemployed with regards to health literacy as well as the methods that were applied to assess these needs in previous studies. To reduce the risk of missing studies of potential interest, the search will be conducted to include studies focusing on the health literacy-related needs of the unemployed in general rather than only the long-term unemployed. The review questions are:

1. What health information needs have been identified among the long-term unemployed?
2. What health-related knowledge gaps have been identified among the long-term unemployed?

3. What health literacy components have been identified as being low among the long-term unemployed?
4. What methods were used to identify the health information needs, the health-related knowledge gaps and/or the health literacy components that are low among the long-term unemployed?

Item 5. Protocol and registration

The protocol will be prospectively registered at the Center for Open Science (OSF): https://doi.org/10.17605/OSF.IO/PW8MX. The study adheres to the PRISMA-ScR guidelines for scoping reviews (Tricco et al., 2018; refer to the Appendix).

Item 6. Eligibility criteria

The eligibility criteria for this scoping review are based on the PCC (Population, Concept and Context) criteria recommended for scoping reviews:

Inclusion criteria:

1. Study designs: Primary studies with any designs
2. Publication status: published in peer-reviewed journals or other sources (e.g., project reports, organisational reports, dissertations/theses)
3. ***P: Population***: Persons officially registered as unemployed, looking for employment or participating in programs aimed at reintegration into the workforce
4. ***C: Concept***: Health information needs of the unemployed, their health-related knowledge gaps as well as the components of health literacy that have been observed to be low in this population group
5. ***C: Context***: Non-clinical populations in non-clinical settings

Exclusion criteria:

1. No primary data: literature reviews, letters, editorials, comments, book reviews, monographs
2. Studies in clinical settings and/or with clinical samples
3. Health literacy not assessed and/or not reported
4. Other target populations than unemployed persons or population not defined
5. Participants not working due to other reasons (homemakers, chronically ill, retired)
6. Conference abstracts

7. Other language than English or German
8. Full-text not accessible

Item 7. Information sources

The following bibliographic databases will be searched from inception to January 2021:

1. MEDLINE via OvidSP
2. CINAHL via EBSCO
3. PsycINFO via EBSCO
4. Social Sciences Citation Index (SSCI) via Clarivate
5. SCOPUS via Elsevier
6. Applied Social Sciences Index and Abstracts (ASSIA) via ProQuest

To identify grey literature, a search for sources relevant to this review will be conducted in websites of relevant national and international institutions. The reference lists of all sources included in the scoping review will be screened for further sources of interest.

Item 8. Search

The search will be conducted in English. No language or date limitations will be set at the search stage. We aim to include sources in English or in German. The search strategy will be developed iteratively by the team that includes a professional librarian. The search terms will reflect the PCC criteria relevant for this scoping review:

Population (Unemployment) *AND* **Concept** (Health literacy)

Item 9. Selection of sources of evidence

The search in the listed databases will be done by the professional librarian on our team and the search results will be saved in the reference management software, Endnote. After deduplication in Endnote, the remaining results will be exported to the literature review software Covidence. Following a further check for duplicates in Covidence, the scoping review will be done in two stages: (i) screening of titles and abstracts and (ii) screening of full texts of the studies included into the next stage. Both stages will be done independently by two reviewers based on the inclusion and exclusion criteria set for this scoping review. Any discrepancies will be resolved during discussion until consensus is reached.

Item 10. Data charting process

A data-charting form[1] will be developed for this scoping review and calibrated in the team. The team will discuss and agree upon how data items will be selected and the software to be used and will test and refine the charting form to ensure that all relevant data will be captured. The full data charting will be conducted independently by two team members. Any discrepancies will be discussed until consensus is reached.

Item 11. Data items

The following data items for data-charting will be coded:

1. Generic bibliographic information
2. Design and study aims
3. Characteristics of examined population
4. Measuring health literacy
5. Characteristics of needs assessment
6. Findings and limitations

Item 12. Critical appraisal of individual sources of evidence

As part of the overview of heath literacy needs of the unemployed, we aim to map the characteristics and quality of methods used to assess the needs, including whether the health topics focussed on were determined in consultation with the participants or pre-set by the researchers. As our search is bound to identify various sources of evidence, including quantitative, qualitative and mixed-methods approaches, we will not use a specific assessment tool, but will focus on a selection of questions addressing aspects such as selection of study participants, aim of the study (whether to improve health literacy or for work reintegration purposes), methods used to assess health literacy, determination of health topic focused on and funding sources.

[1] The term 'data charting' rather than 'data extraction' is used in a scoping review to describe the methods of extracting the data from the included sources of evidence (e.g., calibrated or tested forms) and any processes for obtaining and confirming data from investigators (e.g. Arksey and O'Malley, 2005; Tricco et al 2018).

Item 13. Summary measures

Not applicable for scoping reviews.

Item 14. Synthesis of results

A qualitative summary of study characteristics and the health literacy-related needs will be provided based on the information charted as part of the scoping review. If possible, the health literacy-related needs will be grouped according to various demographic characteristics of the participants in the primary studies. The methods used to assess the needs will also be summarised and recommendations for best-practice procedures will be formulated.

Item 15. Risk of bias across studies

Not applicable for scoping reviews.

Item 16. Additional analyses

Not applicable for scoping reviews.

Items 17-26: Results / Discussion

Not applicable at the protocol stage.

Item 27: Funding

German Federal Ministry of Health, grant number ZMVI1-2519FSB020

Appendix: PRISMA extension for scoping reviews (PRISMA-ScR) checklist

Tricco AC, Lillie E, Zarin W, O'Brien KK, Colquhoun H, Levac D, et al. PRISMA extension for scoping reviews (PRISMA-ScR): checklist and explanation. Annals of Internal Medicine. 2018;169(7):467-73. doi:10.7326/M18-0850

Section	Item	PRISMA-ScR Checklist Item
Title	1	Identify the report as a scoping review.
**Abstract**		
Structured summary	2	Provide a structured summary that includes (as applicable): background, objectives, eligibility criteria, sources of evidence, charting methods, results, and conclusions that relate to the review questions and objectives.
**Introduction**		
Rationale	3	Describe the rationale for the review in the context of what is already known. Explain why the review questions/objectives lend themselves to a scoping review approach.
Objectives	4	Provide an explicit statement of the questions and objectives being addressed with reference to their key elements (e.g., population or participants, concepts, and context) or other relevant key elements used to conceptualize the review questions and/or objectives.
**Methods**		
Protocol and registration	5	Indicate whether a review protocol exists; state if and where it can be accessed (e.g., a Web address); and if available, provide registration information, including the registration number.
Eligibility criteria	6	Specify characteristics of the sources of evidence used as eligibility criteria (e.g., years considered, language, and publication status), and provide a rationale.
Information sources	7	Describe all information sources in the search (e.g., databases with dates of coverage and contact with authors to identify additional sources), as well as the date the most recent search was executed.
Search	8	Present the full electronic search strategy for at least 1 database, including any limits used, such that it could be repeated.
Selection of sources of evidence	9	State the process for selecting sources of evidence (i.e., screening and eligibility) included in the scoping review.
Data charting process	10	Describe the methods of charting data from the included sources of evidence (e.g., calibrated forms or forms that have been tested by the team before their use, and whether data charting was done independently or in duplicate) and any processes for obtaining and confirming data from investigators.
Data items	11	List and define all variables for which data were sought and any assumptions and simplifications made.
Critical appraisal	12	If done, provide a rationale for conducting a critical appraisal of

of individual sources of evidence		included sources of evidence; describe the methods used and how this information was used in any data synthesis (if appropriate).
Summary measures	13	*Not applicable for scoping reviews*
Synthesis of results	14	Describe the methods of handling and summarizing the data that were charted.
Risk of bias across studies	15	*Not applicable for scoping reviews*
Additional analyses	16	*Not applicable for scoping reviews*
**Results**		
Selection of sources of evidence	17	Give numbers of sources of evidence screened, assessed for eligibility, and included in the review, with reasons for exclusions at each stage, ideally using a flow diagram.
Characteristics of sources of evidence	18	For each source of evidence, present characteristics for which data were charted and provide the citations.
Critical appraisal within sources of evidence	19	If done, present data on critical appraisal of included sources of evidence (see item 12).
Results of individual sources of evidence	20	For each included source of evidence, present the relevant data that were charted that relate to the review questions and objectives.
Synthesis of results	21	Summarize and/or present the charting results as they relate to the review questions and objectives.
Risk of bias across studies	22	*Not applicable for scoping reviews*
Additional analyses	23	*Not applicable for scoping reviews*
**Discussion**		
Summary of evidence	24	Summarize the main results (including an overview of concepts, themes, and types of evidence available), link to the review questions and objectives, and consider the relevance to key groups.
Limitations	25	Discuss the limitations of the scoping review process.
Conclusions	26	Provide a general interpretation of the results with respect to the review questions and objectives, as well as potential implications and/or next steps.
**Funding**	27	Describe sources of funding for the included sources of evidence, as well as sources of funding for the scoping review. Describe the role of the funders of the scoping review.
