# Supplementary material for: Health Literacy Needs Among Unemployed Persons: Collating Evidence Through Triangulation of Interview and Scoping Review Data
Source: Front Public Health. 2022 Feb 22;10:798797. doi: 10.3389/fpubh.2022.798797 (PMC8902044; doi:10.3389/fpubh.2022.798797)
Supplement: Supplementary file 1 [file Data_Sheet_1.ZIP › Supplementary file 10_Translation of used quotes.pdf]

Supplementary file 10: Translations of quotes used in the manuscript

Health literacy needs among unemployed persons: collating evidence through triangulation of interview and scoping review data

Authors:

Florence Samkange-Zeeb[(1)], Hunny Singh [(2)], Meret Lakeberg [(1,2)], Jonathan Kolschen [(2)], Benjamin Schüz[(2)], Lara Christianson[(1)], Karina Karolina De Santis[(1)], Tilman Brand[(1)], Hajo Zeeb[(1,2)]

[(1)] Leibniz Institute for Prevention Research and Epidemiology – BIPS. Department of Prevention and Evaluation
[(2)] University of Bremen, Faculty of Human and Health Sciences (Public Health)

Corresponding author: Hajo Zeeb, zeeb@leibniz-bips.de, Tel: +49 421 21856902

	German	English
Finding health-related information		
	„Das Problem des Internets ist aber auch, dort habe ich immer ein Aber, weil manchmal übertreiben sie auch. Du liest manchmal, Zum Beispiel: Ich bin betroffen von jenem und ich weiß, wie ich mich fühle, dann schreibe ich das ja da auf. Die nächste Person hat das gleiche, aber beschreibt es anders, weil er sich anders fühlt. Da ist auch die Frage, was glaubt man.“ (Interview 9, male, 39 years)	“But the problem of the internet is also; there I always have a but, because sometimes they also exaggerate. You read sometimes, for example: I have this, and I know how I feel, then I write that down there. The next person has the same, but describes it differently, because he feels differently. That´s also the question: what to believe?” (Interview 9, male, 39 years)

	„Also ich gebe zu, dass ich durch meine Frau viel im Internet und auf YouTube schaue, ehrlich gesagt. Weil es gibt dort nicht nur etwas zu Ernährung, sondern auch alle diese Leute… Influencer. Manchen glaubt man natürlich nicht, weil sie ihre Sachen verkaufen wollen, aber ich denke manche Sachen sind auch echt. Die zeigen, wie man abnehmen kann, wenn man übergewichtig ist, was man essen soll, wie viele Kalorien man zu sich nehmen soll und was man nicht zu sich nehmen soll. Also ich nehme quasi viel aus dem Netz: YouTube, Instagram und Facebook.“ (Interview 9, male, 39 years)	“Well, to be honest, I admit that I watch a lot on the Internet and YouTube because of my wife. Because there is not only something about nutrition, but there are also all these people… influencers. Of course you don´t believe some of them because they want to sell their stuff, but I think some things are also true. They show you how to lose weight if you´re overweight, what to eat, how many calories to eat and what not to eat. So I kind of take a lot from the Internet: YouTube, Instagram and Facebook.” (Interview 9, male, 39 years)
	„Ich gucke vielmehr wegen Ernährung diese Leute an, um Sport zu machen. Weil die erklären dir, wenn du zum Beispiel deine Bauchmuskeln trainieren willst, musst du erstmal das und das essen und für das genaue Ziel das trainieren. Oder wenn du Brust haben willst… Also immer diese Sportler, nicht nur… Ich kann nicht jemanden angucken, der so aussieht (Andeutung mit den Armen) und mir über Ernährung etwas erklärt. Natürlich nicht.“ (Interview 9, male, 39 years)	“I look at these people more because of nutrition, to do sports. Because they explain to you that, if you want to train your abdominal muscles, for example, you first have to eat this and that, and train this for the exact goal. Or if you want to develop your chest muscles (….). Well, always these athletes, not only…. I can´t look at someone who looks like this (indicates an overweight person using arms) and explains to me about nutrition. Of course not.” (Interview 9, male, 39 years)
Accessing healthcare		
	„Hauptsächlich mit Frau Steiner, weil sie ist, glaube ich, diejenige, die das auch beantragt oder mit guckt wie, was möglich ist.“ (Interview 6, female, 36 years)	"I mainly talk to Mrs. S [the social worker] [for advice], because I think she is the one who also applies for [new orthopedic working shoes] or helps check what is possible.” (Interview 6, female, 30 years)
	„Habe ich dann noch mit Frau Steiner gesprochen und versucht Therapeuten wiederzufinden, weil das doch besser wäre, wenn ich einen habe.“ (Interview 6, female, 36 years)	“I talked to Mrs. S [the social worker] and tried to find [with her] a therapist again, because it would be better if I had one.” (Interview 6, female, 36 years)

	„Also ich schätze mal die einfachste Weise… weil sogar bei manchen Ärzten ist es zurzeit… ist Internet halt. Ich kann sagen das Internet. Genau. Obwohl sie keine Ärzte sind, aber im Internet, da hat man wirklich viele Informationen. Ob man das wirklich glaubt oder nicht glaubt, aber die Informationen sind da vorhanden.“ (Interview 9, male, 39 years)	“Well, I guess the easiest way… because even with some doctors it´s currently… is the Internet. I can say the Internet. Exactly. Although they are not doctors, but on the internet, one really has a lot of information there. Whether one really believes it or not, but the information is there.” (Interview 9, male, 39 years)
Use of/interaction with healthcare services		
	“Da frage ich dann doch lieber den Arzt oder den Apotheker. Und das ist auch ein feiner Mann hier, der nimmt sich auch viel Zeit für die Leute, das ist schon mal gut(…). Aber nicht wirklich Rezept raus, rein, auf Wiedersehen und, nein, nein, der redet noch mit den Leuten. Das ist gut. Finde ich richtig geil.“ (Interview 1, male, 53 years	“I'd rather ask the doctor or the pharmacist. And that's also a decent man (The Pharmacist), he also takes a lot of time for the people, that's good (...). Really, not just prescription, out, in, goodbye and…, no, no, he still talks to the people. That's good. I think that is really cool.” (Interview 1, male, 53 years)
	“(…) Der Kurs war leider in den Arbeitszeiten drinnen mit der Trainerin, mit der ich mich auch gut verstanden habe. Das hat dann mit den Arbeitszeiten nicht mehr so geklappt. (…)<br>“ja ich hatte auch den gleichen [Aquafitness-] Kurs bei einer anderen Trainerin mal gemacht (….), die war auch ganz freundlich, das war auch ganz gut aber irgendwie hatte ich zu der nicht so [die gleiche Chemie] (…..) , aber irgendwie fand ich das mit der anderen Trainerin irgendwie besser. (Interview 6, female, 30 years)	“The [aqua fitness] course with the trainer with whom I got along well was unfortunately during my working hours. That didn't work out so well with my working hours. (…)<br>I also once did the same course with another trainer (...), she was also quite friendly, that was also quite good, but somehow I didn't have the same [connection] to her (...), I somehow found it better with the other trainer.” (Interview 6, female, 30 years)
	“Beim Arzt, wie ich es gesagt habe, du gehst dort hin, du kannst das gleiche… zum Beispiel kannst du sagen „ich habe heute Bauchschmerzen“ und du bekommst Paracetamol. Morgen komme ich dort hin und sage ich	“At the doctor's (…) you go there, for example you can say "I have stomachache today" and you get paracetamol. Tomorrow I go there and I say “I have a headache” and I get ibuprofen and paracetamol. They

	habe Kopfschmerzen und bekomme Ibuprofen und Paracetamol. Die geben dir die gleichen Sachen, das ist so. Manchmal sage ich, bevor ich sogar hingehe, ich gehe lieber Paracetamol kaufen. Weil ich weiß, wenn ich hingehe, bekomme ich Paracetamol." (Interview 9, male, 39 years)	give you the same stuff, it's like that. Sometimes before I even go there I say, I'd rather go buy paracetamol. Because I know if I go, I'm going to get paracetamol." (Interview 9, male, 39 years)
	"Ich war vor zwei Jahren mal da, dann hatte ich eine Kehlkopfentzündung. Ich wusste, dass das [kein] normaler Husten ist und der verschreibt mir ACC Akut. Ich sagte ihm auch, dass ich kein ACC Akut brauche und dass das schon länger ist. Ich merke das ja, dass das nicht normal ist. „Ja, dann gebe ich Ihnen eine Überweisung für den HNO-Arzt", sagte er. Und dann bin ich hin und der hat gesagt, ja Kehlkopfentzündung und Antibiotika. Und ich habe dem Arzt noch vorher gesagt „bräuchte ich Antibiotika oder so? (Interview 7, male, 52 years)	"I was there two years ago, I had laryngitis. I knew that it was [not] a normal cough and he would prescribe me ACC Acute. I also told him that I don't need ACC and that it's been going on for a while. I can tell this is not normal. "Okay, then I'll give you a referral for the ENT specialist," he said. And then I went and he said, yes, laryngitis and antibiotics. And I had told the doctor before "don't I need antibiotics or something?" (…) and he gave me ACC." (Interview 7, male, 52 years)
Application of health information		
	„Unter der Woche ist das ein bisschen schlecht, weil abends habe ich keine Lust zu kochen und hier auf der Arbeit gibt es ja kaum Gemüse. Ich kann mir das selber holen, aber wir essen ja mittags wenig Gemüse, sage ich mal." (Interview 7, male, 52 years)	"During the week it's not so good, because in the evening I don't feel like cooking and here at work [in the canteen] there are hardly any vegetables." (Interview 7, male, 52 years)
	„Ich bin dann kaputt [nach der Arbeit], weil ich auch körperlich arbeite. Wie gesagt, dann gibt es eine Kleinigkeit zu essen, nicht immer gesundes. Wenn es mal schnell gehen soll, gibt es eine Dose, aber es gibt immer den Apfel dazu." (Interview 10, male, 54 years)	"I am exhausted [after work] because I also work physically. As already said, then there's something small to eat, not always healthy. When it has to be quick, it's a can [of food], but there is always an apple with it." (Interview 10, male, 54 years)

	“Aber ich sehe zu, dass ich irgendwie versuche, wie gesagt, mich vollwertig zu ernähren. Das Gute ist hier ist ich habe dazu die Bewegung, also Bewegung und Sport gehören eigentlich immer dazu, aber ich bin hier so eingespannt, dass ich am Wochenende irgendwie auch… Aber ich habe so meinen ganz guten… Ganz gut ausgewogene Momente, wo dieses sich Ernähren, Verarbeiten, die Nahrung, Bewegen - ohne jetzt anzusetzen - ich bin Diabetiker. Ich bin mal mehr oder mal weniger diszipliniert. Ich weiß wie es geht, ich habe eine Diabetiker-Schulung hinter mir, manchmal sündige ich. Dann habe ich aber auch wieder einen Tag, wo ich, was weiß ich, hundert mal Treppen steigen muss. Ich versuche immer das so ein bisschen auszubalancieren und das gelingt mir so ganz gut.“ (Interview 10, male, 54 years)	“But I try somehow, as already said, to keep a well-balanced diet. The good thing here is that I have the exercise, so exercise and sports are actually always part of it, but I'm so busy here that during the weekend I somehow also... But I do have my quite good... quite well balanced moments, where this eating, processing, the food, moving - without putting on weight - I'm diabetic. I am sometimes more or sometimes less disciplined. I know how it goes, I took part in a diabetic training course, I sometimes sin. But then again I have a day where I have to, I don't know, climb stairs a hundred times. I always try to balance things a bit and that works out quite well for me.”(Interview 10, male, 54 years)
	“Ja theoretisch ist alles möglich bei mir aber praktisch ist ein bisschen schlecht. Beispiel also ich denke mal, ich müsste mich eigentlich mehr bewegen aber ich tu es nicht. Das ist einfach so jetzt der Satz. Interviewer: Also es ist eher mehr theoretisch, als es in der Praxis? Teilnehmer: Ja weil ich weiß es ja, weil ich bin ja nun auch keine 20 mehr und mit 20 hat man gewisse Dinge einfach noch nicht gewusst und jetzt mit Ü50 weiß man dann schon mehr und deswegen ist, es hapert einfach an der Umsetzung. Warum kann ich jetzt gar nicht so beantworten.“ (Interview 5, female, 55 years)	“Well, theoretically everything is possible for me, but practically it is not so good. So, for example, at times I think I should actually be more physically active, but I don't do anything about it. That’s the simple sentence.<br>Well, because I do know, because I'm not 20 anymore and with 20 one simply didn't know certain things and now with over 50 one does know more and that's why, the problem is just the implementation. I can't really answer why.” (Interview 5, female, 55 years)
	„An der Ernährung selber habe ich ein bisschen versucht oder versuche ich immer ein bisschen nicht zu viel Zucker oder zu viel fettige Sachen zu nehmen, aber	"I've tried [to work] a little bit on nutrition itself, or I always try a bit not to eat too much sugar or too much fatty stuff, but when I really notice that I'm not feeling

	wenn ich wirklich merke, mir geht es so seelisch gerade nicht so gut, dann esse ich das, was mir gerade schmeckt. Ob das jetzt der triefendste, fettigste Burger ist oder so.“ (Interview 6, female, 30 years	so good mentally at the moment, then I eat whatever tastes good to me at that time. Be it the soggiest, greasiest burger or whatever.” (Interview 6, female, 30 years)
