# Supplementary material for: Health Literacy Needs Among Unemployed Persons: Collating Evidence Through Triangulation of Interview and Scoping Review Data
Source: Front Public Health. 2022 Feb 22;10:798797. doi: 10.3389/fpubh.2022.798797 (PMC8902044; doi:10.3389/fpubh.2022.798797)
Supplement: Supplementary file 1 [file Data_Sheet_1.ZIP › Supplementary file 2_MEDLINE search strategy.pdf]

Supplementary file 2: MEDLINE search strategy

Health literacy needs among unemployed persons: collating evidence through triangulation of interview and scoping review data

Authors:

Florence Samkange-Zeeb[1], Hunny Singh [2], Meret Lakeberg [1,2], Jonathan Kolschen [2], Benjamin Schüz[2], Lara Christianson[1], Karina Karolina De Santis[1], Tilman Brand[1], Hajo Zeeb[1,2]

[1] Leibniz Institute for Prevention Research and Epidemiology – BIPS. Department of Prevention and Evaluation
[2] University of Bremen, Faculty of Human and Health Sciences (Public Health)

Corresponding author: Hajo Zeeb, zeeb@leibniz-bips.de, Tel: +49 421 21856902

Database: MEDLINE(R) and Epub Ahead of Print, In-Process & Other Non-Indexed Citations, Daily and Versions(R)
Date range: 1946 to January 20, 2021
Platform: Ovid
Search date: January 21, 2021

Search line	Query
1	exp "health literacy"/
2	"health literacy".ti,ab.
3	readability.ti,ab.
4	"health knowledge".ti,ab.
5	"health competenc*".ti,ab.
6	"medical literacy".ti,ab.
7	((information or literacy or knowledge) adj2 (seek* or find* or obtain* or understand* or process* or capacit* or skill or skills)).ti,ab.
8	((health or medical) adj2 (literac* or knowledge or competenc* or skill*)).ti,ab.
9	or/1-8
10	exp unemployment/
11	unemploy*.ti,ab.
12	redundan*.ti,ab.
13	jobless.ti,ab.

14	laid-off.ti,ab.
15	"laid off".ti,ab.
16	workless.ti,ab.
17	((out or lost or loss or lose) adj2 (work* or job* or employ*)).ti,ab.
18	((job* or employment* or work*) adj2 (seek* or search* or look*)).ti,ab.
19	or/10-18
20	and/9,19
