# Supplementary material for: Health Literacy Needs Among Unemployed Persons: Collating Evidence Through Triangulation of Interview and Scoping Review Data
Source: Front Public Health. 2022 Feb 22;10:798797. doi: 10.3389/fpubh.2022.798797 (PMC8902044; doi:10.3389/fpubh.2022.798797)
Supplement: Supplementary file 1 [file Data_Sheet_1.ZIP › Supplementary file 3_COREQ checklist.pdf]

Supplementary file 3: COREQ checklist

Health literacy needs among unemployed persons: collating evidence through triangulation of interview and scoping review data

Authors:

Florence Samkange-Zeeb[(1)], Hunny Singh [(2)], Meret Lakeberg [(1,2)], Jonathan Kolschen [(2)], Benjamin Schüz[(2)], Lara Christianson[(1)], Karina Karolina De Santis[(1)], Tilman Brand[(1)], Hajo Zeeb[(1,2)]

(1) Leibniz Institute for Prevention Research and Epidemiology – BIPS. Department of Prevention and Evaluation

(2) University of Bremen, Faculty of Human and Health Sciences (Public Health)

Corresponding author: Hajo Zeeb, zeeb@leibniz-bips.de, Tel: +49 421 21856902

Consolidated criteria for reporting qualitative studies (COREQ): 32-item checklist [1]

**No. Item**	**Guide questions/description**	**Reported on Page #**
**Domain 1: Research team and reflexivity**		
Personal Characteristics		
1. Interviewer/facilitator	Which author/s conducted the interview or focus group?	Methods, page 5: Supplementary file
2. Credentials	What were the researcher's credentials? E.g. PhD, MD	Supplementary file 4
3. Occupation	What was their occupation at the time of the study?	Supplementary file 4
4. Gender	Was the researcher male or female?	Supplementary file 4
5. Experience and training	What experience or training did the researcher have?	Supplementary file 4
Relationship with participants		

6. Relationship established	Was a relationship established prior to study commencement?	Methods, page 4/5: Supplementary file 4
7. Participant knowledge of the interviewer	What did the participants know about the researcher? e.g. personal goals, reasons for doing the research	Methods, page 5: Supplementary file 4
8. Interviewer characteristics	What characteristics were reported about the inter viewer/facilitator? e.g. Bias, assumptions, reasons and interests in the research topic	Supplementary file 4
**Domain 2: study design**		
Theoretical framework		
9. Methodological orientation and Theory	What methodological orientation was stated to underpin the study? e.g. grounded theory, discourse analysis, ethnography, phenomenology, content analysis	Methods, page 5
Participant selection		
10. Sampling	How were participants selected? e.g. purposive, convenience, consecutive, snowball	Methods, page 5
11. Method of approach	How were participants approached? e.g. face-to-face, telephone, mail, email	Methods, page 5
12. Sample size	How many participants were in the study?	Results
13. Non-participation	How many people refused to participate or dropped out? Reasons?	N/A
Setting		
14. Setting of data collection	Where was the data collected? e.g. home, clinic, workplace	Methods, page 5
15. Presence of non-participants	Was anyone else present besides the participants and researchers?	Methods, page 5
16. Description of sample	What are the important characteristics of the sample? e.g. demographic data, date	Results, page 7
Data collection		
17. Interview guide	Were questions, prompts, guides provided by the authors? Was it pilot tested?	Methods page 5; Supplementary file 5
18. Repeat interviews	Were repeat inter views carried out? If yes, how many?	Methods, page 5
19. Audio/visual recording	Did the research use audio or visual recording to collect the data?	Methods, page 5
20. Field notes	Were field notes made during and/or after	N/A

	the interview or focus group?	
21. Duration	What was the duration of the interviews or focus group?	Methods, page 5
22. Data saturation	Was data saturation discussed?	N/A
23. Transcripts returned	Were transcripts returned to participants for comment and/or correction?	N/A
**Domain 3: analysis and findings**		
Data analysis		
24. Number of data coders	How many data coders coded the data?	Methods, page 5
25. Description of the coding tree	Did authors provide a description of the coding tree?	Supplementary file 7
26. Derivation of themes	Were themes identified in advance or derived from the data?	Methods, page 5
27. Software	What software, if applicable, was used to manage the data?	QCAmap[2]
28. Participant checking	Did participants provide feedback on the findings?	Strengths and limitations, page 14
Reporting		
29. Quotations presented	Were participant quotations presented to illustrate the themes/findings? Was each quotation identified? e.g. participant number	Results, pages 8-12
30. Data and findings consistent	Was there consistency between the data presented and the findings?	Results, pages 7-12
31. Clarity of major themes	Were major themes clearly presented in the findings?	Results, pages 7-12
32. Clarity of minor themes	Is there a description of diverse cases or discussion of minor themes?	Discussion, pages 12-14

1. Tong A, Sainsbury P, Craig J. (2007). Consolidated criteria for reporting qualitative research (COREQ): a 32-item checklist for interviews and focus groups. Int J Qual Health Care. 19(6):349-57. doi:10.1093/intqhc/mzm042
2. Fenzl, T., & Mayring, P. (2017). QCAmap: eine interaktive Webapplikation für Qualitative Inhaltsanalyse. *Zeitschrift für Soziologie der Erziehung und Sozialisation, 37*, 333-339. Software available: https://www.qcamap.org/ [Last accessed 20.10.2021]
