# Supplementary material for: Health Literacy Needs Among Unemployed Persons: Collating Evidence Through Triangulation of Interview and Scoping Review Data
Source: Front Public Health. 2022 Feb 22;10:798797. doi: 10.3389/fpubh.2022.798797 (PMC8902044; doi:10.3389/fpubh.2022.798797)
Supplement: Supplementary file 1 [file Data_Sheet_1.ZIP › Supplementary file 4_Researcher team and reflexivity.pdf]

Supplementary file 4: Research team and reflexivity

Health literacy needs among unemployed persons: collating evidence through triangulation of interview and scoping review data

Authors:

Florence Samkange-Zeeb[1], Hunny Singh [2], Meret Lakeberg [1,2], Jonathan Kolschen [2], Benjamin Schüz[2], Lara Christianson[1], Karina Karolina De Santis[1], Tilman Brand[1], Hajo Zeeb[1,2]

[1] Leibniz Institute for Prevention Research and Epidemiology – BIPS. Department of Prevention and Evaluation
[2] University of Bremen, Faculty of Human and Health Sciences (Public Health)

Corresponding author: Hajo Zeeb, zeeb@leibniz-bips.de, Tel: +49 421 21856902

Characteristic	Description
Credentials	HZ and BS are professors. HZ is also an MD. FSZ, KDS and TB hold doctoral degrees. HS is a doctoral candidate. ML and JK are Public Health students. LC is a professional librarian.
Occupation	FSZ, ML, HZ, LC, KDS and TB work at the Leibniz Institute for Prevention Research and Epidemiology -BIPS: FSZ, KDS and TB as senior researchers, ML as a student assistant, HZ as a Head of Department, and LC as a librarian. HZ is also a professor of Public Health at the University of Bremen. HS, JK and BS work at the University of Bremen: HS as a researcher, JK as a student assistant, and BS as a professor of Public Health and Head of Department.
Gender	FSZ, ML, LC and KDS are female; HS, JK, BS, HZ and TB are male.
Experience and training	FSZ, BS, HZ, KDS and TB are public health scientists with many years of experience in health research. They have all co-authored multiple publications using qualitative and/or quantitative research methods. LC is an experienced professional librarian.
Relationship between participants and	The researchers did not know any of the

researchers	participants prior to the study. FSZ, JK, BS and HZ comprised the team that presented the project to participants of a workforce re-integration program run by a partner institution of the FORESIGHT project at study onset. Three of the persons who attended the presentation took part in the interviews conducted by FSZ and HS, as well as in the workshop.
Participant knowledge of the interviewer	The participants knew that the interviewers were researchers at the University of Bremen/Leibniz Institute for Prevention Research and Epidemiology - BIPS. They were provided with written information about the aims of the project. The social workers at the respective reintegration institutions also explained the aims of the project to the participants.
Interviewer characteristics	FSZ and HS's interest in research on health literacy and social inequalities in health formed the basis for their interest in the topic.
