# Supplementary material for: Health Literacy Needs Among Unemployed Persons: Collating Evidence Through Triangulation of Interview and Scoping Review Data
Source: Front Public Health. 2022 Feb 22;10:798797. doi: 10.3389/fpubh.2022.798797 (PMC8902044; doi:10.3389/fpubh.2022.798797)
Supplement: Supplementary file 1 [file Data_Sheet_1.ZIP › Supplementary file 5_Interview guide.pdf]

# Supplementary file 5: Interview guide (English translation on page 3)

Health literacy needs among unemployed persons: collating evidence through triangulation of interview and scoping review data

**Authors:**

Florence Samkange-Zeeb[1], Hunny Singh [2], Meret Lakeberg [1,2], Jonathan Kolschen [2], Benjamin Schüz[2], Lara Christianson[1], Karina Karolina De Santis[1], Tilman Brand[1], Hajo Zeeb[1,2]

[1] Leibniz Institute for Prevention Research and Epidemiology – BIPS. Department of Prevention and Evaluation

[2] University of Bremen, Faculty of Human and Health Sciences (Public Health)

**Corresponding author:** Hajo Zeeb, zeeb@leibniz-bips.de, Tel: +49 421 21856902

**Original, German version**

**Einleitend:** Wir möchten mit Ihnen heute über Gesundheit sprechen und dabei besonders auf Themen achten, die Ihnen wichtig sind. Uns interessiert auch, wie Sie mit Informationen zu Gesundheit umgehen.

- Wenn Sie an Gesundheit denken, was fällt Ihnen als erstes ein?

- Was ist Ihnen persönlich beim Thema Gesundheit wichtig?
    - Welche Themen sind Ihnen wichtig?
    - Worüber hätten Sie gerne mehr Informationen?
    - Wie wichtig ist Ihnen Gesundheit in Ihrem Alltag?
    - Gibt es andere Bereiche, die gerade wichtiger sind als Gesundheit?

- Was machen Sie, wenn Sie ein Gesundheitsproblem haben?
    - Mit wem besprechen Sie ihre Gesundheitsprobleme?
    - Wer unterstützt Sie, wenn Sie Gesundheitsprobleme haben?
    - Was macht es leichter oder schwieriger für Sie, Unterstützung für Ihr Gesundheitsproblem zu bekommen?

- Wie einfach ist es für Sie an Gesundheitsinformation zu kommen?
    - Wo suchen Sie sich Informationen zu den Themen, die sie interessieren?
    - Mit wem sprechen Sie, wenn Sie gesundheitliche Informationen brauchen?

    - Was macht es leichter oder schwieriger für Sie, an diese Informationen zu kommen?
    - Welchen Informationen trauen Sie, welchen eher nicht?
    - Woran machen Sie das fest?

- Wie sollen Informationen am besten zur Verfügung gestellt werden? Wie sollen sie eher nicht bereitgestellt werden?

- Wie gut verstehen Sie, was Ihr Arzt/Ihre Ärztin Ihnen sagt?
    - Was machen Sie, wenn sie Ihren Arzt/Ihre Ärztin nicht verstehen?

- Nehmen Sie an Präventionsmaßnahmen/Angebote wie Zahnuntersuchungen, Rückenkurse o.ä. teil?

Jetzt denken Sie an Ihre Kollegen und Kolleginnen:

- Was ist Ihren Kollegen/Kolleginnen beim Thema Gesundheit wichtig?
- Woher bekommen/suchen Ihre Kollegen/Kolleginnen Information zum Thema Gesundheit?

<u>Wenn Gesundheitsdienstleistungen in Anspruch genommen werden:</u>

- Welche Dinge gefallen Ihnen gut, wenn Sie zum Arzt oder zu einer Ärztin gehen?
- Welche Dinge gefallen Ihnen nicht so gut, wenn Sie zum Arzt oder zu einer Ärztin gehen?

**Translated English version:**

**Introduction:** Today we would like to talk to you about health and especially focus on topics that are important to you. We are also interested in how you look for and use health information.

- When you think about health, what is the first thing that comes to your mind?
    - What is important to you personally regarding the topic health?
    - Which topics are important to you?
    - What would you like to have more information on?
    - How important is health for you in your daily life?
    - Are there other areas that are currently more important than health?

- What do you do when you have a health problem?
    - Who do you discuss your health problems with?
    - Who supports you when you have health problems?
    - What makes it easier or more difficult for you to get support when you have a health problem?

- How easy is it for you to get health information?
    - Where do you look for information on the topics that interest you?
    - Who do you talk to when you need health information?
    - What makes it easier or more difficult for you to get this information?
    - Which information do you trust, and which do you distrust?
    - How do you make this decision?

- How should information be best made available? How should it rather not be made available?

- How well do you understand what your doctor says to you?
    - What do you do when you don't understand your doctor?

- Do you take part in preventive measures/offers such as dental check-ups, exercise courses for your back, etc.?

Now think about your colleagues:
- What is important to your colleagues regarding the topic health?
- Where do your colleagues get/search for information on health?

<u>If health care services are used:</u>
- What kind of things do you like when you go to the doctor~~‘s~~?
- What kind of ~~-~~things do you dislike when you go to the doctor~~’s~~?
