# Supplementary material for: Health Literacy Needs Among Unemployed Persons: Collating Evidence Through Triangulation of Interview and Scoping Review Data
Source: Front Public Health. 2022 Feb 22;10:798797. doi: 10.3389/fpubh.2022.798797 (PMC8902044; doi:10.3389/fpubh.2022.798797)
Supplement: Supplementary file 1 [file Data_Sheet_1.ZIP › Supplementary file 6_Research questions.pdf]

Supplementary file 6: List of research questions

Health literacy needs among unemployed persons: collating evidence through triangulation of interview and scoping review data

Authors:

Florence Samkange-Zeeb[(1)], Hunny Singh [(2)], Meret Lakeberg [(1,2)], Jonathan Kolschen [(2)], Benjamin Schüz[(2)], Lara Christianson[(1)], Karina Karolina De Santis[(1)], Tilman Brand[(1)], Hajo Zeeb[(1,2)]

(1) Leibniz Institute for Prevention Research and Epidemiology – BIPS. Department of Prevention and Evaluation

(2) University of Bremen, Faculty of Human and Health Sciences (Public Health)

Corresponding author: Hajo Zeeb, zeeb@leibniz-bips.de, Tel: +49 421 21856902

**Health topics of interest to participants/aspects participants associate with 'health'**
Description of health topics interviewees are interested in
Description of factors/aspects that interviewees associate with the term "health"
Description of health topics interviewees say are of interest to their colleagues
**Finding and appraising health-related information**
Description of sources from which interviewees get health-related information
Description of source(s) from which interviewees prefer to get health-related information
Description of barriers interviewees face when wanting to access health-related information
Description of factors that facilitate interviewees' access to health-related information.
Description of how interviewees think health information should be made available
Description of how interviewees deal with Internet information
Do interviewees generally understand the health information they get/access?
Description of what interviewees do when they don't understand the information they get
Description of sources from which colleagues/friends get health information
**Accessing healthcare**
Description of what interviewees do when they have a health problem
Description of barriers interviewees face when wanting to access healthcare services
Description of factors that facilitate interviewees' access to healthcare
Description of how use of health services can be made easier (from perspective of interviewees)
Description of support structures of interviewees when faced with health problems
**Use of services/interaction with doctor**
Description of how interviewees react when they don't understand what their doctor says to them.

Which prevention measures (check-ups) do interviewees take part in?<br>Reasons why interviewees do not take part in prevention measures (check-ups)<br>Do interviewees generally understand what their doctor says to them?<br>Description of satisfaction with doctor<br>Description of what interviewees do when not satisfied with doctor
**Application of health knowledge**<br>Extent to which interviewees apply the health-related information they possess<br>Description of what interviewees do (after working hours) to maintain/improve their health
