# Supplementary material for: Health Literacy Needs Among Unemployed Persons: Collating Evidence Through Triangulation of Interview and Scoping Review Data
Source: Front Public Health. 2022 Feb 22;10:798797. doi: 10.3389/fpubh.2022.798797 (PMC8902044; doi:10.3389/fpubh.2022.798797)
Supplement: Supplementary file 1 [file Data_Sheet_1.ZIP › Supplementary file 7_Coding frame example.pdf]

Supplementary file 7: Coding frame example

Health literacy needs among unemployed persons: collating evidence through triangulation of interview and scoping review data

Authors:

Florence Samkange-Zeeb[1], Hunny Singh [2], Meret Lakeberg [1,2], Jonathan Kolschen [2], Benjamin Schüz[2], Lara Christianson[1], Karina Karolina De Santis[1], Tilman Brand[1], Hajo Zeeb[1,2]

[1] Leibniz Institute for Prevention Research and Epidemiology – BIPS. Department of Prevention and Evaluation
[2] University of Bremen, Faculty of Human and Health Sciences (Public Health)

Corresponding author: Hajo Zeeb, zeeb@leibniz-bips.de, Tel: +49 421 21856902

Question for analysis: Description of sources from which interviewees get health-related information

Inductive category formation was used as content analytical technique. In accordance with the definition of the selection criterion, sources of information on which interviewees said they relied were coded as answers. Sources of information mentioned or referred to by the interviewees throughout the transcript were coded.

Hauptkategorie	Subkategorie	Beispiel/Zitate
Offizielle Gesundheitsanbieter	Arzt/Ärztin	"Naja gut, in erster Linie würde ich eventuell die Arztpraxis, auch zum Hausarzt und der wird mir ja dann irgendetwas sagen.", "Ja, also bei mir ist meine Informationsquelle direkt beim Arzt."
	Apotheke/r/in	"Nun, ich brauche doch bloß in die Apotheke zu gehen und dann bin ich informiert [...]. Da sind die Informationen in so einer Dings, da kannst du sie dir rausnehmen, mit nachhause nehmen, da liest du dir das durch in Ruhe."

	Gynäkologe/in	"Ich hatte zum Beispiel eine Frauenärztin, mit der hatte ich auch ganz ehrlich erzählt, dass ich die Male davor diesen Test eben nicht gemacht habe. Und da hat sie mir eben auch empfohlen, dass ich ihn wirklich mal machen sollte jetzt. Und dann habe ich mir auch gesagt so, so, das machst du jetzt sofort, du gibst den auch gleich sofort ab. Ja, und das war eben auch dann im Nachhinein wichtig gewesen, dass ich es gemacht habe. Und jetzt habe ich mir eben für die Zukunft überlegt, ja, das eben dann auch regelmäßig zu machen."
	Krankenkasse	"Einmal hatte ich wegen so Kurse geguckt bei der AOK."
	Dermatologe/in	"Auch von einer Hautärztin."
	Gesundheitsamt	"Da würde ich dann beim Gesundheitsamt anrufe."
Familie und Freunde	Partner/ Familie/ Freunde	"Die ist ausgebildete Krankenschwester, die weiß auch noch ein bisschen was, das ist zwar ein bisschen länger her […]. Die unterstützt dann auch manchmal."
	Mutter	"Ja, also ich sage jetzt mal so, vielleicht mehr meine Mutter. Weil bei der kriege ich immer am meisten mit, was sie schon hat. Weil sie ja immer älter wird, ist ganz klar. Und ja, worauf sie so achtet, dass kriege ich dann am meisten mit."
	Bekannte	"Von einer Kundin aus dem Laden [habe ich das gehört]."
Internet/digitale Medien	Social media	"Also ich gucke normalerweise viele Medien. Ich bin ehrlich. Medien halt.", "Deshalb habe ich früher nur eine Sportlerseite angeguckt mit den Ernährungstipps von der Sportlerseite. Und dann sagen sie dir wie viele Portionen am Tag man essen soll, lieber viel, bei manche essen einmal viel und manche essen 5-6 mal am Tag.",
	Internet	"Ja ich würde im Internet gucken […] oder ja das kommt immer ganz darauf an, was ich möchte.", "Also ich schätze mal die einfachste Weise, weil sogar bei manchen Ärzten ist es zurzeit, ist Internet halt. Ich kann sagen das Internet. Genau. Obwohl sie keine Ärzte sind, aber im Internet, da hat man wirklich viele Informationen.", "Man kann sich ja alles übers Internet holen, sage ich jetzt mal [...]. In dem Falle, wo ich dann zur Darmspiegelung musste, das war so das erste Mal, wo ich dann nachgelesen habe was, wie soll ich das jetzt so sagen, dass es nicht immer das schlimmste sein muss, wenn man zur Darmspiegelung geht.", "Bei meiner Freundin gucke ich dann im Internet nach."

	Chatforen	"Also ich gehe auch schon mal in Chatforum gucken oder also jetzt selber mich nicht anmelden, aber halt wirklich so die Probleme oder eventuell Lösungen, die die haben und gucke dann eben, wenn die das Problem von sich beschreiben, ob das mit meinen gleichkommt oder ob ich mich damit identifizieren kann, dann gucke ich halt irgendetwas, wenn andere Nutzer dazu geschrieben, was denen helfen konnte oder so."
	YouTube	"Also man wird ja bombardiert von jeder Ecke, also wenn man das Thema Ernährung eingibt bei YouTube, da hast du so vieles: Wollen Sie abnehmen, sind Sie übergewichtig, auf was man achten soll, weißt du? Ich interessiere mich für alles – ich gucke alles."
	Influencer	"Genau, Influencer. Manchen glaubt man natürlich nicht, weil sie ihre Sachen verkaufen wollen, aber ich denke manche Sachen sind auch echt. Die zeigen, wie man abnehmen kann, wenn man übergewichtig ist, was man essen soll, wie viele Kalorien man zu sich nehmen soll und was man nicht zu sich nehmen soll."
	Instagram/ Facebook	"Also ich nehme quasi viel aus dem Netz: YouTube, Instagram und Facebook."
Arbeitsplatz	Sozialarbeiter am Arbeitsplatz	"Ich habe Vertrauen zu meiner Sozialarbeiterin hier als Informationsquelle.", "Hier [die Sozialarbeiterin] Frau S. zum Beispiel, die erzählt ja auch manchmal etwas. Wir hatten ja zum Beispiel den Gesundheitstag, 1-2 mal im Jahr glaube ich, und da gibt es auch immer Informationen. Und sie erzählt auch zwischendurch mal irgendetwas.", "Ich würde auch Frau S. fragen zum Beispiel als allererstes, wenn ganz akut was ist oder ich absolut nicht wüsste, wen ich fragen kann, dann frage ich sie, wen kann ich fragen und die sagt mir das dann, so."
	Kollegen/ Kolleginnen	"Mit meiner Kollegin [spreche ich über Gesundheit].
	Arbeitsplatz	"Hin und wieder passiert das ja hier mal, irgendwelche Sachen mit dem Rücken, wie man den Rücken entlastet bei der Arbeit."
	Coaching am Arbeitsplatz	"Dann hatten wir auch dieses Coaching und dann hatte ich auch eine Frau hier […]. Jeder hatte sein eigenes Coaching und ich hatte mich für Entspannung entschieden."
TV/Nachrichten/Zeitschriften	Magazin der Krankenkasse	"Ich kriege ja jedes Mal von der Krankenkasse immer Hefte zugeschickt[…]. Ja durchlesen und dann wieder entsorgen."

	Zeitungen/ Zeitschriften	"Oder kann auch lesen in den ganzen Zeitungen, in den Zeitschriften.", "In den ganzen Zeitschriften, da steht ja auch überall immer so viel drin [...]. Ja diese, die es so am Kiosk gibt, sage ich jetzt mal so. Nicht die Tageszeitung, sondern so die Zeitschriften, wo über Prominente auch was drinsteht und sowas. [...] Also sowas [über Gesundheit] steht immer drin."
	TV/Radio	"Im Fernsehen gibt es ja auch immer so vieles, was man so hört.", "Morgen[s] NTV, ich fahre jeden Tag Auto und höre jeden Tag nur Corona, Corona.", "NTV, N24.", "SAT1 lass' ich mich immer informieren."
	Nachrichten	"Über die Nachrichten oder über soziale Netzwerke, so wie bei Facebook, aber am meisten über die Nachrichten."
Sonstiges	Friseur	"Ich war bei […] Friseuren, was weiß ich, warum die [Haare] auf einmal so, dünner geworden sind und ausgefallen sind."
