# Supplementary material for: Health Literacy Needs Among Unemployed Persons: Collating Evidence Through Triangulation of Interview and Scoping Review Data
Source: Front Public Health. 2022 Feb 22;10:798797. doi: 10.3389/fpubh.2022.798797 (PMC8902044; doi:10.3389/fpubh.2022.798797)
Supplement: Supplementary file 1 [file Data_Sheet_1.ZIP › Supplementary file 8_Scoping_Review_excluded after full-text.pdf]

## Supplementary file 8: List of peer-reviewed publications excluded from the scoping review at full-text screening stage

Health literacy needs among unemployed persons: collating evidence through triangulation of interview and scoping review data


**Authors:**

Florence Samkange-Zeeb[(1)], Hunny Singh [(2)], Meret Lakeberg [(1,2)], Jonathan Kolschen [(2)], Benjamin Schüz[(2)], Lara Christianson[(1)], Karina Karolina De Santis[(1)], Tilman Brand[(1)], Hajo Zeeb[(1,2)]

(1) Leibniz Institute for Prevention Research and Epidemiology – BIPS. Department of Prevention and Evaluation

(2) University of Bremen, Faculty of Human and Health Sciences (Public Health)

**Corresponding author:** Hajo Zeeb, zeeb@leibniz-bips.de, Tel: +49 421 21856902


| **Author, year & Title of the article** | **Reason for exclusion** |
|---|---|
| **Alfano-Sobsey et al. 2014** – Community health needs assessment in Wake County, North Carolina: partnership of public health, hospitals, academia, and other stakeholders [1] | Study population not unemployed/not defined |
| **Baumann et al. 2017** – Who benefits from computer based brief alcohol intervention? Day-to-day drinking patterns as a moderator of intervention efficacy [2] | Health literacy not assessed and/or not reported |
| **Eftekhari et al. 2014** – Mental health priorities in Iranian women: overview of social determinants of mental health [3] | Study population not unemployed/not defined |
| **Ernstmann et al. 2020** – DNFV Memorandum Health Literacy (Part 1) – Background, Relevance, Research Topics and Question in Health Services Research [4] | Not a primary study |
| **French et al. 1987** – The East Community Health Project [5] | Study population not unemployed/not defined |
| **Gele et al. 2016** – Health literacy: the missing link in improving the health of Somali immigrant women in Oslo [6] | Study population not unemployed/not defined |
| **Halbert et al. 2010** – Participation rated and representativeness of African Americans recruited to a health promotion program [7] | Health literacy not assessed and/or not reported |
| **Heitor dos Santos et al. 2017** – Promotion of mental health literacy and mental well-being in a Portuguese unemployed population sample: Effectiveness assessment of a capacity building community-based intersectoral intervention [8] | Conference abstract |
| **Snodgrass et al. 2020** – Evaluation of a culturally sensitive social and emotional well-being program for Aboriginal and Torres Strait Islanders [9] | Study population not unemployed/not defined |

| | |
|---|---|
| **Hergenrather et al. 2013** – A pilot test of the HOPE Intervention to explore employment and mental health among African American gay men living with HIV/AIDS: results from a CBPR study [10] | Clinical setting |
| **Hoffman et al. 1997** – Women´s health status and use of health services in a rapidly growing peri-urban area of South Africa [11] | Focus on general population |
| **Hollederer et al. 2019** – Unmet Medical Need in Germany: Analyses of EU-SILC-Survey from 2005 to 2014 [12] | Focus on general population |
| **Israel et al. 1994** – Health Education and Community Empowerment: Conceptualizing and Measuring Perception of Individual, Organizational, and Community Control [13] | Not a primary study |
| **Jafari 2010 et al. 2010** – Knowledge of determinants of mental health among Iranian immigrants of BC, Canada: "a qualitative study" [14] | Study population not unemployed/ not defined |
| **Javanparast et al. 2020** – Health service access and utilization amongst culturally and linguistically diverse population in regional South Australia – qualitative study [15] | Study population not unemployed/not defined |
| **Kennedy 2001** – Community Involvement at What Cost? – Local Appraisal of a Pan-European Nutrition Promotion Programme in Low-Income Neighbourhoods [16] | Health literacy not assessed and/or not reported |
| **Kilpatrick et al. 1985** – Life-Styles and Psychological Well-Being Among Unemployed Men in Northern Ireland [17] | Health literacy not assessed and/or not reported |
| **Kreuzfeld et al. 2013** – Health effects and acceptance of a physical activity program for older long-term unemployed workers [18] | Health literacy not assessed and/or not reported |
| **Murty et al. 2009** – Policies/programs for reducing health inequalities by tackling nonmedical determinants of health in the United Kingdom [19] | Not a primary study |
| **Ness et al. 2018** – Empowering the local community through a flexible education program [20] | Conference abstract |
| **Niedorys et al. 2020** – Health Literacy – a review of research using the European Health Literacy Questionnaire (HLS-EU-Q16) in 2010-2018 [21] | Not a primary study |
| **Nogueira et al. 2016** – Nutrition Label Numeracy: Disparities and Association with Health Behaviors [22] | Focus on general population |
| **O`Meara et al. 2019** – Low Health Literacy is Associated with Risk of Developing Type 2 Diabetes in a Nonclinical Population [23] | Focus on general population |
| **Omar et al. 2017** – Putting your feet in gloves designed for hands: Horn of Africa Muslim men perspectives in emotional wellbeing and access to mental health services in Australia [24] | Study population not unemployed/not defined |
| **Rowe et al. 1987** – The Fresno County Refugee Health Volunteer Project: a case study in cross-cultural health care delivery [25] | Study population not unemployed/not defined |
| **Rütten et al. 2009** – Assets for policy making in health promotion: Overcoming political barriers inhibiting women in difficult life situations to access sport facilities [26] | Health literacy not assessed and/or not reported |
| **Ryan 2019** – Exploring the experiences of MĀORI men in a culturally enriched well-being programme [27] | Full-text not accessible |
| **Schopper et al. 2000** – When Providers and Community Leaders Define Health Priorities: The Results of Delphi Survey in the Canton of Geneva [28] | Study population not unemployed/not defined |
| **Stafford et al. 1980** – Employment, Work involvement and Mental Health in Less Qualified Young people [29] | Health literacy not assessed and/or not reported |
| **Staiger et al. 2016** – Stigma and poor mental health literacy as | Full-text not accessible |

| | |
|---|---|
| barriers to service use among unemployed people with mental illness – a qualitative study [30] | |
| **Virgolino et al. 2017** – Facing unemployment: study protocol for the implementation and evaluation of a community-based intervention for psychological well-being promotion [31] | Study protocol |
| **Woodall et al. 2014** – Moving prison health promotion along: towards an integrative framework for action to develop health promotion and tackle the social determinants of health [32] | Study population not unemployed/not defined |

## References


1. Alfano-Sobsey, E., et al., *Community health needs assessment in Wake County, North Carolina: partnership of public health, hospitals, academia, and other stakeholders.* N C Med J, 2014. **75**(6): p. 376-83.
2. Baumann, S., et al., *Who benefits from computer-based brief alcohol intervention? Day-to-day drinking patterns as a moderator of intervention efficacy.* Drug Alcohol Depend, 2017. **175**: p. 119-126.
3. Eftekhari, M.B., et al., *Mental health priorities in Iranian women: overview of social determinants of mental health.* Iranian journal of psychiatry, 2014. **9**(4): p. 241-247.
4. Ernstmann, N., et al., *[DNVF Memorandum Health Literacy (Part 1) - Background, Relevance, Research Topics and Questions in Health Services Research].* Gesundheitswesen, 2020. **82**(7): p. e77-e93.
5. French, J. and F. Cunning, *The East Cumbria Community Health Project.* International Quarterly of Community Health Education, 1987. **8**(3): p. 213-218.
6. Gele, A.A., et al., *Health literacy: the missing link in improving the health of Somali immigrant women in Oslo.* BMC Public Health, 2016. **16**(1): p. 1134.
7. Halbert, C.H., et al., *Participation rates and representativeness of African Americans recruited to a health promotion program.* Health Education Research, 2009. **25**(1): p. 6-13.
8. Heitor dos Santos, M.J., et al., *Promotion of mental health literacy and mental well-being in a Portuguese unemployed population sample: Effectiveness assessment of a capacity building community-based intersectoral intervention.* European Psychiatry, 2017. **41**(S1): p. S736-S736.
9. Snodgrass, W.J., et al., *Evaluation of a culturally sensitive social and emotional well-being program for Aboriginal and Torres Strait Islanders.* Australian Journal of Rural Health, 2020. **28**(4): p. 327-337.
10. Hergenrather, K.C., et al., *A Pilot Test of the HOPE Intervention to Explore Employment and Mental Health Among African American Gay Men Living With HIV/AIDS: Results From a CBPR Study.* AIDS Education and Prevention, 2013. **25**(5): p. 405-422.
11. Hoffman, M., et al., *Women's health status and use of health services in a rapidly growing peri-urban area of South Africa.* Social Science & Medicine, 1997. **45**(1): p. 149-157.
12. Hollederer, A. and M. Wildner, *[Unmet Medical Need in Germany: Analyses of EU-SILC-Survey from 2005 to 2014].* Dtsch Med Wochenschr, 2019. **144**(1): p. e1-e11.

13. Israel, B.A., et al., *Health education and community empowerment: conceptualizing and measuring perceptions of individual, organizational, and community control.* Health education quarterly, 1994. **21**: p. 149-70.
14. Jafari, S., S. Baharlou, and R. Mathias, *Knowledge of Determinants of Mental Health Among Iranian Immigrants of BC, Canada: "A Qualitative Study".* Journal of Immigrant and Minority Health, 2010. **12**(1): p. 100-106.
15. Javanparast, S., S.K.A. Naqvi, and L. Mwanri, *Health service access and utilisation amongst culturally and linguistically diverse populations in regional South Australia: a qualitative study.* Rural Remote Health, 2020. **20**(4): p. 5694.
16. Kennedy, L., *Community involvement at what cost?—Local appraisal of a pan-European nutrition promotion programme in low-income neighbourhoods.* Health promotion international, 2001. **16**: p. 35-45.
17. Kilpatrick, R. and K. Trew, *Life-styles and psychological well-being among unemployed men in Northern Ireland.* Journal of Occupational Psychology, 1985. **58**: p. 207-216.
18. Kreuzfeld, S., et al., *Health effects and acceptance of a physical activity program for older long-term unemployed workers.* International Archives of Occupational and Environmental Health, 2013. **86**(1): p. 99-105.
19. Murty, S., et al., *Policies/Programs for Reducing Health Inequalities by Tackling Nonmedical Determinants of Health in the United Kingdom*.* Social Science Quarterly, 2009. **90**(5): p. 1403-1422.
20. Ness, A., et al., *Empowering the local community through a flexiible education program.* International Journal of Integrated Care. (s1):21, 2018.
21. Niedorys, B., A. Chrzan-Rodak, and B. Ślusarska, *Health Literacy – a review of research using the European Health Literacy Questionnaire (HLS-EU-Q16) in 2010-2018.* Pielegniarstwo XXI wieku / Nursing in the 21st Century, 2020. **19**(1): p. 29-41.
22. Nogueira, L.M., et al., *Nutrition Label Numeracy: Disparities and Association with Health Behaviors.* Am J Health Behav, 2016. **40**(4): p. 427-36.
23. O'Meara, L., et al., *Low Health Literacy Is Associated With Risk of Developing Type 2 Diabetes in a Nonclinical Population.* The Diabetes Educator, 2019. **45**(4): p. 431-441.
24. Omar, Y.S., J. Kuay, and C. Tuncer, *'Putting your feet in gloves designed for hands': Horn of Africa Muslim men perspectives in emotional wellbeing and access to mental health services in Australia.* International Journal of Culture and Mental Health, 2017. **10**(4): p. 376-388.
25. Rowe, D.R. and H.P. Spees, *The Fresno County Refugee Health Volunteer Project: a case study in cross-cultural health care delivery.* Migr World Mag, 1987. **15**(4): p. 22-7.
26. Rütten, A., et al., *Assets for policy making in health promotion: Overcoming political barriers inhibiting women in difficult life situations to access sport facilities.* Social Science & Medicine, 2009. **69**(11): p. 1667-1673.
27. Ryan, T., *Exploring the experiences of Maori men in a culturally enriched well-being programme.* Kai Tiaki Nursing Research, 2019. **10(1)**: p. 22–28.
28. Schopper, D., et al., *When providers and community leaders define health priorities: the results of a Delphi survey in the canton of Geneva.* Soc Sci Med, 2000. **51**(3): p. 335-42.
29. Stafford, E.M., P.R. Jackson, and M.H. Banks, *Employment, work involvement and mental health in less qualified young people.* Journal of occupational Psychology, 1980. **53**(4): p. 291-304.

30. Staiger, T., et al., *Stigma and Poor Mental Health Literacy As Barriers to Service Use Among Unemployed People With Mental Illness – A Qualitative Study.* European Psychiatry, 2016. **33**(S1): p. S487-S487.
31. Virgolino, A., et al., *Facing unemployment: study protocol for the implementation and evaluation of a community-based intervention for psychological well-being promotion.* BMC Psychiatry, 2017. **17**(1): p. 261.
32. Woodall, J., et al., *Moving prison health promotion along: towards an integrative framework for action to develop health promotion and tackle the social determinants of health.* Criminal Justice Studies, 2014. **27**(1): p. 114-132.
