# Supplementary material for: Health Literacy Needs Among Unemployed Persons: Collating Evidence Through Triangulation of Interview and Scoping Review Data
Source: Front Public Health. 2022 Feb 22;10:798797. doi: 10.3389/fpubh.2022.798797 (PMC8902044; doi:10.3389/fpubh.2022.798797)
Supplement: Supplementary file 1 [file Data_Sheet_1.ZIP › Supplementary file 9_Full-text data extracted.pdf]

Supplementary file 9: Characteristics and summary of findings of publications included in the scoping review

Health literacy needs among unemployed persons: collating evidence through triangulation of interview and scoping review data

Authors:

Florence Samkange-Zeeb[(1)], Hunny Singh [(2)], Meret Lakeberg [(1,2)], Jonathan Kolschen [(2)], Benjamin Schüz[(2)], Lara Christianson[(1)], Karina Karolina De Santis[(1)], Tilman Brand[(1)], Hajo Zeeb[(1,2)]

[(1)] Leibniz Institute for Prevention Research and Epidemiology – BIPS. Department of Prevention and Evaluation
[(2)] University of Bremen, Faculty of Human and Health Sciences (Public Health)

Corresponding author: Hajo Zeeb, zeeb@leibniz-bips.de, Tel: +49 421 21856902

Characteristics and summary of findings of the 5 peer-reviewed and one grey literature source included in the scoping review

Author, year, country	Study design/aims/outcomes	Participant characteristics	Health Literacy (HL) measurement/needs assessment	Findings and limitations
Pertillä et al., 2010, Finland	Cross-sectional survey<br><br>Aim: To study information seeking practices and coping	N=750 long-term unemployed people in Finland (unemployed for more than one year);	Participants asked how often they sought information about health<br><br>Measurement on a scale from 1	-High mix-focused copers were most active informationseekers concerning both unemployment and health<br>-Connection between coping functions and

	strategies of long- term unemployed people. Additionally, connection between coping functions and everyday life information seeking is studied<br><br>Data collection via questionnaires sent home in 2007<br><br>Outcomes: information seeking behavior, coping strategies for unemployment<br><br>Data analysis method: Descriptive analyses, connection between coping functions and information seeking studiedby cross tabulating coping functions with information seeking	recruitment through ministry of labor; 73% over 54<br><br>Names of respondents receivedthrough Ministry of Labor, questionnaire and 1 reminder sent between October/November 2007	(tryingto avoid such information) to 6 (very often), scale was further divided into: seldom, occasionally and often<br><br>Characteristics of needs assessment<br><br>-Questionnaires used to determine needs<br><br>-Needs identified regarding information seeking connected with unemployment and information seeking about health	information seeking practices of the long-term unemployed determined<br>-High problem-focused copers significantly more active inorienting information seeking than medium and low problem-focused copers<br>-In problem-specific information seeking, those who use alot of mixed-focused coping proved to be most active<br><br>Limitations: none reported by the authors, self-reportedoutcomes, selection bias (750 of 1600 long-term unemployed replied), especially interested may have replied<br><br>Conclusion: Connection between coping functions andinformation seeking likely<br><br>No information regarding funding and conflict of interestgiven
Santos et al., 2018, Portugal	Delphi technique (2 rounds)<br><br>Aim: To create expert consensus regarding how to develop and implement an intervention program for mental health promotion among unemployed people<br><br>Experts contacted by E-Mail in, data collected through structured questionnaire (Delphi technique) in 2015	N=46 experts for mental health/ employment/temporary work/ prevention of psychiatric disorders recruited in Portugal by snowball sampling method; Average age: 48,17+-12.48<br><br>Further characteristics: psychologists (34.8%), psychiatrists (17.4%), sociologists (15.2%), doctorate degree (47.8%)	-MHL defined as identifying signs and symptoms of depression, anxiety and stigma regarding mental health<br>-Answers regarding importance of contents and skills to promote by the intervention collected through 5-point Likert scale (1= totally disagree and 5 totally agree)<br><br>Characteristics of needs assessment<br><br>-Delphi technique entailing experts from various backgrounds	Important intervention components identified:<br>-promotion of MHL (mainly about anxiety, mood disorders and stigma about mental health),<br>-methods to challenge unemployment (as promotion of job searching skills through job-interviewing training)<br>-mental health promotion skills (self-regulation of emotions, effective communication training, awareness of skills and personal facets)<br>-favorised structure: small groups (up to 10 participants) on more than 10 weekly sessions (each 2 hours)<br><br>Main outcomes to be measured:

	Outcomes: Consensual items for a mental health intervention among unemployed people (models/paradigms of intervention, contents of interventions, frequency/duration of sessions, size of groups, intervention setting)<br><br>Data analysis: Descriptive analyses for each Delphi round (mean values, coefficients of variation, Standard deviation by mean), consensus flag variables were created		-Consensus obtained throughout 2 rounds of data collection	-participants satisfaction with intervention<br>-indicators of mental health (as anxiety or general psychosocial functioning)<br>- strong focus on Portuguese population<br><br>Limitations: unemployed people not asked directly,<br>46/75 contacted experts participated, results influenced by perception of those who participated
Staiger et al., 2017, Germany	Semi-structured interviews<br><br>Aim: To identify barriers to and facilitators of help-seeking and service use based on experiences of unemployed people with mental health problems<br><br>Participants recruitment in 2014/15 from employment agencies and social organisations via handouts and flyers<br><br>Outcome: Experience with help-seeking and mental health service use with a focus on barriers and facilitators	N=15 (7 female, 8 male) unemployed people with self- reported psychological distress, in Germany, between 19 and 63 (mean age 48 years). 53% divorced, 80% born in Germany, one third single, 60% living alone, 47% university entrance diploma, 27% depressive. Unemployment period between 2 months and 15 years<br><br>Recruitment via employment agencies and social organisations via handouts and flyers	HL assessed as knowledge-related facilitators and barriers of service use, questions included:<br><br>What do you know about mental health and its prevention? If you had a mental illness where would you seek help or where and how do you find information regarding mental health<br><br>Participants interviewed individually for 30 to 100 minutes<br><br>Characteristics of needs assessment<br><br>In-depth interviews, needs assessed regarding mental HL, stigma and discrimination experiences and structures and conditions of health	Main barriers of help-seeking: -fear of side effects of psychopharmacological treatment (low MHL)<br>-ineffective psychiatric help<br>-perceived discrimination by mental health care professionals<br>-stigma in the social environment<br>-GPs lack of interest in mental health problems<br>Main Facilitators of help-seeking: -gaining knowledge as motivation factor for treatment<br>-awareness and acceptance of illness<br>-GP as facilitator and supporter<br>-positive relationship between patient and therapist<br><br>Limitations: -participant inclusion not based on self- reported mental health problems and unemployment<br>-80% German citizens, not possible to examine

	Data analysis: Transcripts analysed using qualitative content analysis, interviews coded by 2 independent researchers, MAXQDA 11 used for data analysis		care	differences between ethnic groups<br><br>Conclusion: Unemployed people face barriers as low MHL and benefit from facilitators as awareness knowledge and acceptance of mental health illness<br><br>Funded by German Research Foundation, no conflict of interest reported
Waldmann et al., 2020, Germany	Cross-sectional survey<br><br>Aim: To investigate the influence of MHL on help-seeking intentions and behaviors in unemployed people with mental health issues<br><br>Data collection 2015/16 via questionnaire<br><br>Outcomes: MHL, depression-related knowledge and attitudes toward treatment and treatment options<br><br>Data analysis: Cronbach´s alpha and McDonald´s omega as indices of scale reliability. Associations between MHL variables and help-seeking determined with exploratory factor analysis, pearson correlations of MHL and demographic variables with help-seeking variables, linear	N=301 (50.2% female) unemployed people, mean age average 43.7 years, with mental health problems in Germany. Average unemployment period 35.5 months, mean years of education 13.7.<br><br>Study advertised at unemployment agencies in Ulm, Germany, short phone screening to check eligibility	Mental Health Knowledge Schedule (MAKS) (items scored from 1/strongly disagree to 5/strongly agree/ with don´t know rated as neutral=3, higher sum scores indicating better knowledge, M=22.4, SD=3.1) (ranging from 6-30)<br>Depression Literacy Scale - Statements about effectiveness of depression treatments as true or false, one point for each correct answer, higher scores indicated better literacy, M=3.2, SD=1.8 (from 0-8)<br>Depression with Suicidal Thoughts Vignette (captures ability to recognize depression and suicidal thoughts with a vignette of a 30-year old, participants were supposed to recognize the depressive symptoms and to rate helpfulness of 13 treatment options.<br>Sum score calculated by assigning one point for correct recognition of depression or suicidal thoughts and one for each intervention correctly	-Higher MHL associated with increased help-seeking intentions and behaviors among unemployed individuals with mental health problems<br>-Path analysis showed that different MHL components contribute to help-seeking.<br><br>Limitations: -cross sectional data limit conclusions about causality<br>-participants volunteered for this study may have had better MHL than other unemployed individuals with mental health problems.<br>-2 out of 3 MHL scales focused on depression. All MHL measures showed low reliability, although this can partly be explained by the fact that for example the MAKS was not meant to function as a scale<br><br>Conclusion: Future research should examine the efficacy of MHL interventions to improve help-seeking, especially among vulnerable populations such as the unemployed with increased need for health and social services.<br><br>Funded by German Research Foundation, no conflict of interest reported

	regressions on help-seeking intentions and logistic regressions on help-seeking behaviors		recognized. M=5.5, SD=2.1 (from 0-11)<br><br>General Help-Seeking Questionnaire (GHSQ) - Current intentions to seek help for mental health problems on a 7- point-Likert scale from 1 (very unlikely) to 7 (very likely)<br><br><u>Characteristics of needs assessment</u><br>-3 different scales used to assess various aspects of MHL + GHSQ<br>-Types of needs identified: MHL, knowledge and attitudes specific to depression and ability to recognize depression and suicidal thoughts<br>Items included: knowledge regarding help- seeking, illness recognition, professional support, employment, knowledge and attitudes specific depression	
Wigand et al., 2019, Germany	Longitudinal study<br><br><u>Aim</u>: To assess predictors of help-seeking among unemployed people with mental health problems<br><br><u>Data collection</u> via questionnaire n 2015/16, participants asked in the 6-month f/u if they started new mental health treatment within 6 months after baseline<br><br><u>Outcomes</u>: Barriers and	Baseline: N=301 (50.2% female) unemployed people on average<br>43.7 years with mental health problems in Germany. Average time for unemployment 35.5 months, mean years of education 13.7.<br><br>6-month f/u: n=270 (50.7% female), mean age 44 years, average time for unemployment	MHL assessed using the 8 treatment-related items of the 22-item Depression Literacy scale<br><br>Depressive symptoms were measured using the Patient Health Questionnaire: range of possible sum scores 0–27; M=14.7, SD=5.2; Cronbach's alpha 0.82.<br><br>Frequency of symptoms assessed over the last 2 weeks (from 'not at all'/0 to 'nearly every day'/3).<br><br>Example of items:	In different models<br>-female gender (OR: 1.82; 95% CI: 0.97-1.02)<br>-more depressive symptoms (OR: 1.08, 95% CI: 1.02-1.14)<br>-more MHL (OR: 1.22; 95% CI: 1.03-1.46)<br>-fewer non-stigma-related barriers (OR: 0.28; 95% CI: 0.12-0.63)<br>-mental health service use at baseline (OR: 3.44; 95% CI: 1.57-7.57)<br>significantly predicted new help-seeking during follow-up period<br><br><u>Limitations</u>: Results can´t be generalized since participants were recruited in a region with a

	predictors of help- seeking, MHL, depressive symptoms, beginning of mental health treatment within 6 months after baseline survey<br><br><u>Data analysis</u>: Descriptive analyses and stepwise multiple logistic regression on help-seeking within 6 months after baseline	36.4 months, mean years of education 13.8.<br><br>Study advertised at unemployment agencies, short phone screening to check eligibility	Feeling tired or having little energy or little interest or pleasure in doing things	very low unemployment (3% at that time)<br>Follow-up participants only asked by starting new mental health treatment, existing treatment not reassessed<br><br><u>Conclusion:</u> Intervention should target attitudinal and address especially those outside the mental health care system<br><br>Funded by German Research Foundation, no conflicts of interest reported
Wieland & Hammes, 2010, Germany	Cross-sectional survey<br><br><u>Aim</u>: To explore health literacy and the abilities of German citizens to cope with illnesses (question part of a larger health report)<br><br><u>Data collection</u> via online questionnaire in 2010<br><br><u>Outcomes</u>: demographic characteristics, health literacy, psychological health type, health knowledge, health behavior, work characteristics	N=4764 members of the BARMER GEK health insurance company, among them: N=1417 unemployed people, age mean 61.3 years, 58.8% women; 41.2% men<br><br>Lower secondary education: 6.6%; secondary school: 29.4%; (Advanced technical certificate/ "Fachhochschulreife": 12.4%; high school diploma: 17.1%; university degree: 18.6%; Other 3.5%<br><br>Recruitment: not reported	HL determined via 10 different questions developed by Wieland & Hammes (2008). All questions ranked on a scale of 0-5 (0=it´s not the case at all, 1=is rarely the case, 2= is the case sometimes, 3=is often the case, 4=is very often the case)	-Compared to the employed people the unemployed people had a higher HL (2.53 vs 2.61) – neither age or gender explained the differences<br>-Compared to employed people unemployed participants show lower health knowledge.<br>-Unemployed people spent more time weekly for health behavior<br>-Expectations concerning success in staying healthy received the lowest score and may therefore be regarded as an area of need for an intervention<br>-High HL leads to a better health state<br>-Participants with lower health literacy also spent lower time in health-related activities<br><br><u>Limitations</u>: unemployed population included retired people<br><br><u>Conclusion</u>: The study showed that individual health literacy can be measured as the ability to cope with illness and maintain health<br><br>Funding: BARMER GEK

MHL= Mental health literacy; HL= Health literacy; GP= General practitioner; M=Mean; SD=Standard deviation; OR: Odd ratio; CI: Confidence interval; follow-up=f/u

References:

Perttilä, R., & Ek, S. (2010). Information Behaviour and Coping Functions of Long-Term Unemployed People in Finland. *Libri, 60*, 107-116. doi:10.1515/libr.2010.010

Santos, O., Lopes, E., Virgolino, A., Stefanovska-Petkovska, M., Dinis, A., Ambrosio, S., & Heitor, M. J. (2018). Defining a brief intervention for the promotion of psychological well-being among unemployed individuals through expert consensus. *Frontiers in Psychiatry, 9*. doi:http://dx.doi.org/10.3389/fpsyt.2018.00013

Staiger, T., Waldmann, T., Rusch, N., & Krumm, S. (2017). Barriers and facilitators of help-seeking among unemployed persons with mental health problems: a qualitative study. *BMC health services research*, *17*(1), 39. doi:https://dx.doi.org/10.1186/s12913-017-1997-6

Waldmann, T., Staiger, T., Oexle, N., & Rusch, N. (2020). Mental health literacy and help-seeking among unemployed people with mental health problems. *J Ment Health, 29*(3), 270-276. doi:10.1080/09638237.2019.1581342

Wieland, R. & Hammes, M. (2008). Gesundheitskompetenz als personale Ressource. In K. Mozygemba, S. Mümken, U. Krause et. al (Hrsg.), NutzerInnenorientierung – ein Fremdwort in der Gesundheitssicherung? Bern: Huber- Verlag.

Wieland, R., & Hammes, M. (2010). *Gesundheitsreport 2010 Teil 2 Ergebnisse der Internetstudie zur Gesundheitskompetenz*.

Wigand, M. E., Oexle, N., Waldmann, T., Staiger, T., Becker, T., & Rusch, N. (2019). Predictors of help-seeking in unemployed people with mental health problems. *The International journal of social psychiatry*, *65*(7-8), 543-547. doi:https://dx.doi.org/10.1177/0020764019868262
